# Monitoring the Structure Evolution of Titanium Oxide Photocatalysts: From the Molecular Form via the Amorphous State to the Crystalline Phase

**DOI:** 10.1002/chem.202101117

**Published:** 2021-07-09

**Authors:** Ezgi Onur Şahin, Yitao Dai, Candace K. Chan, Harun Tüysüz, Wolfgang Schmidt, Joohyun Lim, Siyuan Zhang, Christina Scheu, Claudia Weidenthaler

**Affiliations:** ^1^ Heterogeneous Catalysis Max-Planck-Institut für Kohlenforschung Kaiser-Wilhelm-Platz 1 45470 Mülheim an der Ruhr Germany; ^2^ Materials Science and Engineering School for Engineering of Matter Transport and Energy (SEMTE) Arizona State University AZ 85287-8706 Tempe USA; ^3^ Nanoanalytics and Interfaces Max-Planck-Institut für Eisenforschung GmbH Max-Planck-Straße 1 40237 Düsseldorf Germany; ^4^ Department of Chemistry Kangwon National University 24341 Chuncheon Republic of Korea

**Keywords:** crystallization, nanoparticles, PDF (pair distribution function), photocatalysis, reduced titania

## Abstract

Amorphous Ti_x_O_y_ with high surface area has attracted significant interest as photocatalyst with higher activity in ultraviolet (UV) light‐induced water splitting applications compared to commercial nanocrystalline TiO_2_. Under photocatalytic operation conditions, the structure of the molecular titanium alkoxide precursor rearranges upon hydrolysis and leads to higher connectivity of the structure‐building units. Structurally ordered domains with sizes smaller than 7 Å form larger aggregates. The experimental scattering data can be explained best with a structure model consisting of an anatase‐like core and a distorted shell. Upon exposure to UV light, the white Ti_x_O_y_ suspension turns dark corresponding to the reduction of Ti^4+^ to Ti^3+^ as confirmed by electron energy loss spectroscopy (EELS). Heat‐induced crystallisation was followed by in situ temperature‐dependent total scattering experiments. First, ordering in the Ti−O environment takes place upon to 350 °C. Above this temperature, the distorted anatase core starts to grow but the structure obtained at 400 °C is still not fully ordered.

## Graphical abstract

**The formation mechanism** of amorphous Ti_x_O_y_ photocatalysts was studied by X‐ray total scattering experiments and subsequent PDF analysis starting from titanium(IV) ethoxide precursor. Effects of the colour change upon UV illumination on the local Ti environment were investigated by PDF analysis and EELS. Transformation of amorphous Ti_x_O_y_ to anatase‐TiO_2_ by calcination was further examined by in situ temperature dependent experiments.

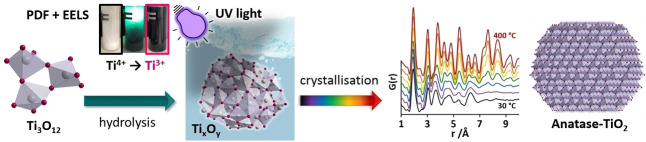

## Introduction

Dating back to the discovery of photocatalytic splitting of water under ultraviolet (UV) light,^[1]^ titanium oxide has been a widely studied photocatalyst. The photocatalytic activity of a material depends on many factors, such as crystal structure, particle size, surface area, exposed crystal facets, and defect density.^[2]^ For high photoactivity, small particle sizes and a low concentration of defects are usually preferred. Such particles benefit from high surface area and facilitate charge transport. In the case of hydrogen generation during water splitting, the generation of excitons as charge carriers with a long lifetime is essential so that they can take part in the water electrolysis half‐reactions.[^3^, ^4^] Mobility and recombination rate of the charge carriers strongly depend on barriers that might exist in the lattice. These include point defects, such as oxygen vacancies, and grain boundaries.[^5^, ^6^] For improving photocatalytic performance, the addition of noble metals as co‐catalysts can support photoactivity if there exists only a low number of active sites. In addition, doping, or introduction of intrinsic defects can decrease the width of the band gap of a given semiconductor photocatalyst.[^5^, ^7^, ^8^, ^9^]

The wide band gap semiconductor TiO_2_ is an economically attractive and environmentally friendly photocatalyst or catalyst support. Moreover, TiO_2_ is also used for many other applications in consumer products, such as a component in toothpaste, sun protection products, or as a food colourant.^[10]^ From the three existing polymorphs brookite, anatase, and rutile, the two latter ones have been studied intensively as photocatalysts. Whereas rutile is the thermodynamically most stable phase,^[11]^ anatase is the stable phase for small crystallites.[^12^, ^13^] It was shown that anatase provides better photocatalytic activity in many photooxidation reactions than rutile.[^14^, ^15^] This was explained by the higher potential of the conduction band minimum relative to the one related to the hydrogen evolution reaction. Other explanations are the higher mobility and longer lifetime of charge carriers in anatase.^[3]^ Compared to rutile, anatase also offers more active surface sites owing to a less dense crystal structure (for anatase ρ_calc_=3.89 g ⋅ cm^−3^ while for rutile ρ_calc_=4.25 g ⋅ cm^−3^)^[15]^ and the ability to generate mobile hydroxyl groups on the surface.^[16]^ Some efforts included the preparation of nanosized “mixed phases” that facilitate efficient electron transfer at the phase boundaries.^[17]^ The commercially available product P25 produced by Evonic has been reported by Hurum et al. as an example to such an attempt.^[17]^ P25 was reported to be composed of mainly anatase (>70 %), smaller amounts of rutile, and a low amount of an amorphous phase, and was generally accepted as reference material for evaluation of photocatalytic activity.[^18^, ^19^] Ohtani et al. on the other hand have suggested more detailed studies to prove the presence or absence of such a synergetic effect originating from the mixed phases.^[19]^

Recently, amorphous titanium oxides have been investigated as photocatalysts.[^20^, ^21^] Surprisingly, their photocatalytic activity was remarkably high compared to the crystalline P25.[^22^, ^23^, ^24^, ^25^, ^26^, ^27^, ^28^, ^29^] However, exploration of the structure‐property relationship of amorphous solids is explicitly challenging due to the limitations of conventional X‐ray methods in analysing structures of amorphous solids. This problem is solved by the application of complementary characterisation techniques, such as extended X‐ray absorption fine structure (EXAFS) or total scattering experiments with subsequent pair distribution function (PDF) analysis. These methods are suitable for analysing local structures at the short‐range order level. Since PDF analysis considers the scattering information of both, Bragg reflections and diffuse scattering, information on the local atomic structure of a compound can be obtained. The latter is not accessible by conventional X‐ray diffraction. Literature studies dedicated to the atomic arrangement in amorphous TiO_2_ materials indicate that the atomic short‐range order depends on synthesis conditions and precursor materials. Petkov et al. published very fundamental work on the atomic structure of amorphous TiO_2_. A combination of electron microscopy, reverse Monte Carlo (RMC) simulations and PDF analysis revealed that the amorphous structure resembles local brookite‐like order.^[30]^ However, the study shows that some synthesis parameters, such as the preparation technique or the type of precursor, can change the ratio of the edge‐ to corner‐sharing octahedra. This, in turn, triggers the transformation to other titanium oxide polymorphs.[^30^, ^31^, ^32^, ^33^, ^34^, ^35^] The atomic PDF of amorphous TiO_2_ obtained via solvothermal synthesis yielded a disordered phase consisting of nanosized layers of connected TiO_6_ octahedra, similar to that in lepidocrocite.[^31^, ^35^] Synthesis of amorphous TiO_2_ is often based on titanium alkoxide precursors. Fernandez‐Garcia et al. examined the local structure of titanium oxide synthesised via the microemulsion method (starting from a titanium alkoxide precursor) by EXAFS and PDF analyses.^[32]^ The nanoparticles exhibited anatase‐like local order with undercoordinated Ti atoms. Temperature‐dependent PDF analysis indicated that the crystallisation of the anatase phase is an intraparticle ordering process, which depends on the surfactant used for the synthesis. Zhang et al. considered size effects of the clusters and derived a structure model that contains an anatase‐like core.^[33]^ Mi et al. analysed the structure of an amorphous titanium oxide synthesised hydrothermally from titanium alkoxide at 250 bar hydrostatic pressure (2 M titanium isopropoxide in isopropanol).^[34]^ They focused on the short‐range arrangements in the amorphous titanium oxide and followed structure changes upon heating to 250 °C by in situ total scattering and PDF analysis. The structure of the amorphous titanium oxide was discussed as titanium hydroxide clusters consisting of TiO_6_/TiO_5_ units with anatase‐like arrangement and OH defects at the surface.

The coloration of TiO_2_‐containing suspensions under UV light exposure has been frequently reported and regarded as evidence of the reduction of Ti centres.[^36^, ^37^, ^38^, ^39^, ^40^, ^41^] Titanium oxide with Ti^3+^ species, or so‐called self‐doped titania,^[42]^ can be obtained by different processes, such as heating under vacuum or reducing atmospheres, plasma‐treating, laser or high‐energy particle bombardment, or exposure to UV light.^[38]^ Presence of Ti^3+^ species together with the effects of changes of electronic properties has been investigated spectroscopically, often by electron paramagnetic resonance (EPR) spectroscopy.[^36^, ^40^, ^43^, ^44^, ^45^, ^46^] Electron microscopy and Raman spectroscopy have been applied to examine structural changes in lattice structures of dark coloured titanium oxides induced upon generation of Ti^3+^ species.[^40^, ^42^, ^47^, ^48^, ^49^] The TiO_2_ catalysts can be synthesised via different routes starting from different precursors. By now, the atomic short‐range structure of amorphous Ti_x_O_y_ reduced under UV illumination has never been investigated by local methods.

The previous investigations have shown that the structures of amorphous TiO_2_ materials can exhibit quite different atomic arrangements. In this work, structural motifs are to be identified and monitored from molecular precursors to the amorphous solid, both with and without exposure to UV light, complemented by a study on heat‐induced crystallisation of the amorphous phase. It was the aim of the systematic study to understand the structure evolution of TiO_2_ starting from the molecular titanium ethoxide precursor, its transformation to the amorphous Ti_x_O_y_ as usually applied in photocatalytic reactions, and finally, the evolution of crystalline polymorphs from the amorphous phase. Potential structure changes induced by illumination of the catalyst with UV light were monitored by X‐ray scattering, transmission electron microscopy (TEM), scanning transmission electron microscopy (STEM), and electron energy loss spectroscopy (EELS). In particular, the local oxidation state of the Ti ions was determined by analysing the electron energy loss near edge structure (ELNES) of each element‐specific edge in the EELS data. The combination of scattering techniques with complementary microscopy experiments completes the information on the structure and the chemical state of the amorphous photocatalysts.

## Results and Discussion

The amorphous Ti_x_O_y_ is synthesised using titanium (IV) ethoxide, Ti(OEt)_4_, as the precursor in the presence of an excess amount of an aqueous solution containing 10 vol % methanol. Upon injection of Ti(OEt)_4_ into the reaction water‐methanol mixture, the precursor goes through hydrolysis, condensation, and polycondensation steps to form partially hydrolysed titanium ethoxide species (h‐Ti_x_O_y_).^[50]^ The X‐ray diffraction (XRD) pattern (Figure S1) collected from the solid after separation and drying (d‐Ti_x_O_y_) does not show any Bragg reflections. For comparison, samples were also synthesised from different precursors, that is, titanium (IV) butoxide [Ti(OBu)_4_] and titanium (IV) isopropoxide [Ti(OiPr)_4_]. The PDFs obtained from the resultant powders are almost identical (Figure S2). This is in accordance with previous work^[22]^ reporting that hydrogen evolution rate, catalyst surface, and morphology were not majorly affected by the choice of titanium alkoxide precursor. Based on this, only materials prepared from Ti(OEt)_4_ were investigated further in this study for local structure analysis as follows (data collected at room temperature except iv):

- pure liquid titanium (IV) ethoxide [Ti(OEt)_4_] in a capillary sealed under protective atmosphere;
- Ti_x_O_y_ synthesised by hydrolysis of Ti(OEt)_4_ and suspended in water/methanol mixture (h‐Ti_x_O_y_);
- Ti_x_O_y_ powder obtained from h‐Ti_x_O_y_ after separation and drying (d‐Ti_x_O_y_);
- d‐Ti_x_O_y_ monitored during in situ temperature‐dependent experiments;
- Ti_x_O_y_ suspension exposed to UV light for 2 h (h‐UV‐Ti_x_O_y_);
- Ti_x_O_y_ powder obtained from h‐UV‐Ti_x_O_y_ after separation and drying (d‐UV‐Ti_x_O_y_).

### Local structure of liquid and crystalline Ti(OEt)_4_ precursors

**Figure 1:**
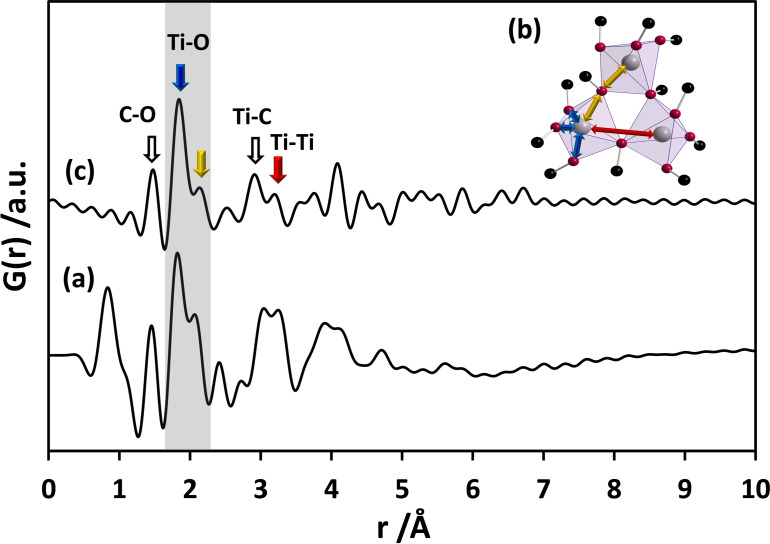
(a) Experimental PDF obtained from the liquid Ti(OEt)_4_ precursor measured in a capillary sealed under protective atmosphere. (b) Ti_3_O_12_C_12_ structure model as given by Ignatyev et al.^[52]^ with additional C atoms (at arbitrary coordinates with fixed C−O bond lengths of 1.47 Å). (c) Simulated PDF for Ti_3_O_12_C_12_. Note that the region representing Ti−O distances is marked in grey. Terminal (short) and bridging (long) Ti−O distances are marked with blue and yellow arrows. Red arrow indicates the distance between Ti−Ti pairs. C−O and Ti−C distances are marked by hollow arrows.

**Figure 2:**
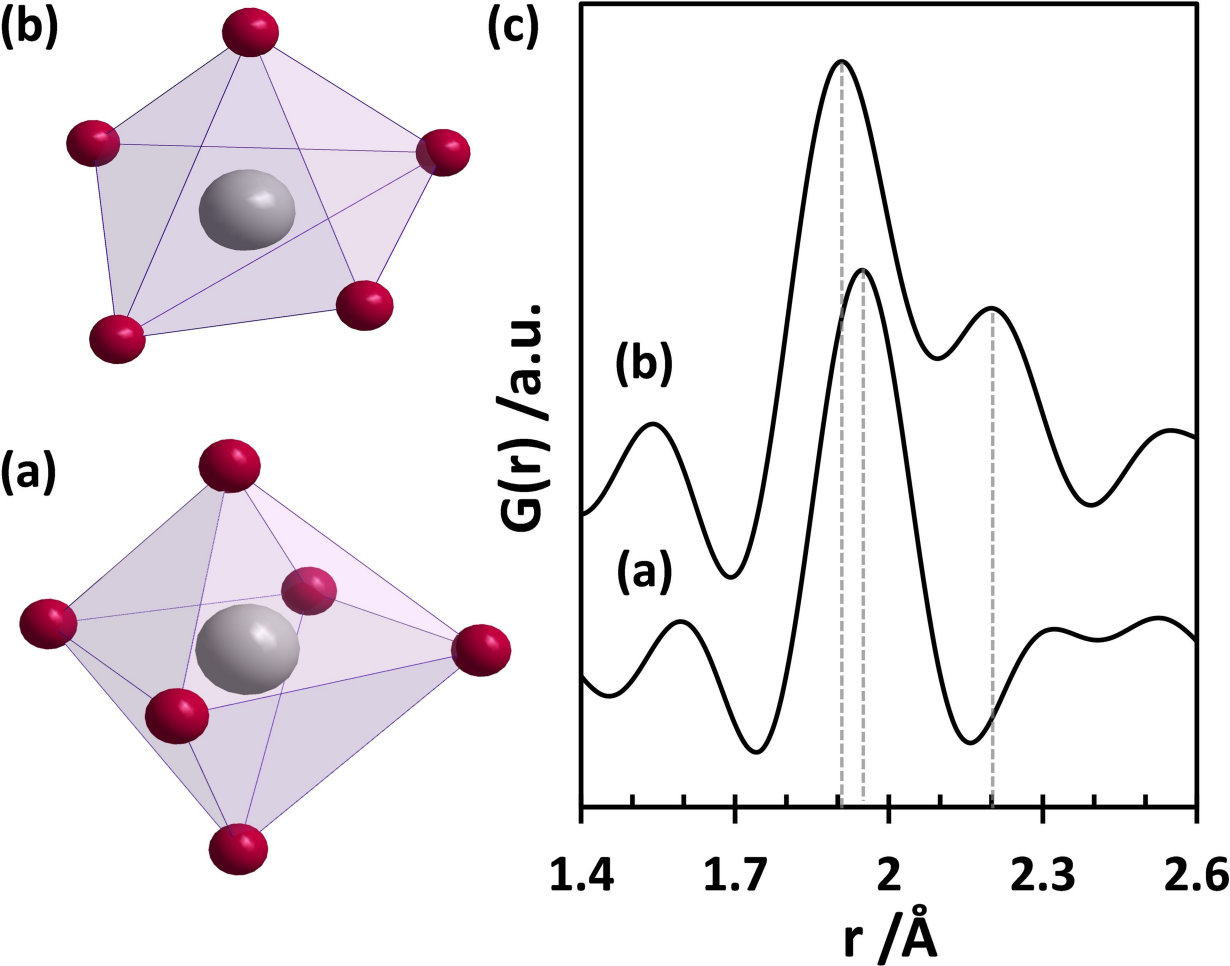
(a) Single TiO_6_ octahedron^[51]^ and (b) single distorted TiO_5_ pentahedron^[33]^ and (c) simulated PDFs for the individual polyhedra. Grey and magenta spheres represent Ti and O atoms. The dotted lines indicate the positions of the maxima of the Ti−O pair distances.

Total scattering experiments and subsequent PDF analysis of the pure liquid titanium ethoxide Ti(OEt)_4_ precursor reveal a pair correlation at 1.45 Å corresponding to the distance of C−O atom pairs in Figure 1a. This peak might also contain contributions from C−C pairs (bond length around 1.54 Å) due to the presence of the ethoxy groups. The most intense peak appears between 1.7–2.3 Å and belongs to Ti−O atom pairs within the Ti−O polyhedra. The primary building units can be either a TiO_6_ octahedron or a TiO_5_ pentahedron.[^33^, ^51^] The simulated PDFs for an isolated octahedron (Figure 2a) and a pentahedron (Figure 2b) help to assign the pair correlations of Ti−O to specific polyhedra. Only one Ti−O distance (at 1.95 Å) is visible for the octahedron while two distinct distances (at 1.91 Å and 2.20 Å) are calculated for the distorted pentahedron. The experimental PDF of the precursor shows one peak at 1.83 Å (bridging) and one belonging to a longer Ti−O distance (terminal) at 2.07 Å. The intensity ratio of these peaks in the experimental PDF is about 3 : 2. The occurrence of two distances supports the presence of pentahedra in the precursor structure.

The structure of titanium ethoxide, Ti(OEt)_4_ has been studied with nuclear magnetic resonance (NMR) spectroscopy, single‐crystal diffraction (XRD), infrared (IR) spectroscopy, X‐ray absorption near‐edge structure (XANES), and extended X‐ray absorption fine structure (EXAFS) analysis.[^53^, ^54^, ^55^, ^56^] From these experimental data, it was concluded that liquid Ti(OEt)_4_ exists as a trimer, Ti_3_(OEt)_12_. The suggested structure models for Ti_3_(OEt)_12_ include cyclic[^54^, ^57^] as well as linear[^58^, ^59^] arrangements of three TiO_6_ octahedra or TiO_5_ polyhedra sharing faces, edges, or corners. Babonneau et al.^[56]^ have proposed that the most realistic structure is the cyclic structure with distorted TiO_5_ pentahedra sharing corners. They also suggested long (9 terminal bonds) and short (6 bridging bonds) Ti−O distances with the ratio of 3 : 2. Considering such a trimeric arrangement, the pair correlations observed at 1.83 and 2.07 Å correspond to the distances between titanium atoms and terminal and bridging oxygen atoms. Ignatyev et al. have calculated an optimised geometry of a trimeric Ti_3_O_12_ species using density functional theory (DFT).^[52]^ Based on the experimental PDF in our study, this model has been used to construct a cluster. Carbon atoms have been added to the oxygen atoms and the C−O bond lengths were fixed to 1.47 Å. The resulting Ti_3_O_12_C_12_ cluster is shown in Figure 1b. The terminal Ti−O bonds (marked by blue arrows in Figure 1b) are reflected by a pair correlation at 1.84 Å, the bridging bonds (marked by yellow arrows in Figure 1b) result in a pair correlation at 2.15 Å (Figure 1c). The measurements confirm an oligomeric structure of the liquid precursor consisting of edge‐sharing pentahedra. Interestingly, the ratio of the bridging to terminal Ti−O pair correlations does not match entirely with the model derived from EXAFS studies and DFT calculations.[^52^, ^56^] The experimental PDF shows a higher number of bridging Ti−O pairs (yellow arrow) and also a higher number of Ti−Ti atom pairs (red arrow). This indicates that the molecular ethoxide precursor exhibits a higher degree of connectivity of the polyhedra, compared to the proposed model, by sharing more corners and edges.

### Local structure of hydrolysed Ti(OEt)_4_ in liquid (h‐Ti_x_O_y_) and in solid state (d‐Ti_x_O_y_)

Hydrolysed h‐Ti_x_O_y_ was obtained by injecting Ti(OEt)_4_ into a mixture of water and methanol as used for the synthesis of the photocatalysts and for photocatalytic water splitting experiments. Following the injection, Ti(OEt)_4_ undergoes hydrolysis, condensation, and polycondensation reactions. The hydrolysis process involves successive addition of Ti−O polyhedra to a cluster by elimination or substitution of the coordinating alkoxy groups.^[60]^ Oligomeric species are formed (oxoethoxides) which display a wide range of nuclearity. The comparison of the PDFs obtained from liquid Ti(OEt)_4_ and its hydrolysed version, h‐Ti_x_O_y_, indicates changes starting from the first Ti−O neighbours (Figure S3). Instead of two distinct Ti−O bond lengths observed for Ti(OEt)_4_, h‐Ti_x_O_y_ shows one slightly broader peak with a maximum at 1.93 Å. After separation of the solid particles from solution and subsequent drying at 60 °C, a powder is obtained which is further on named d‐Ti_x_O_y_. The experimental PDFs of h‐Ti_x_O_y_ and d‐Ti_x_O_y_ are identical within the experimental precision (Figure S4) and therefore the powder sample was used for complementary microscopy and spectroscopy studies.

**Figure 3:**
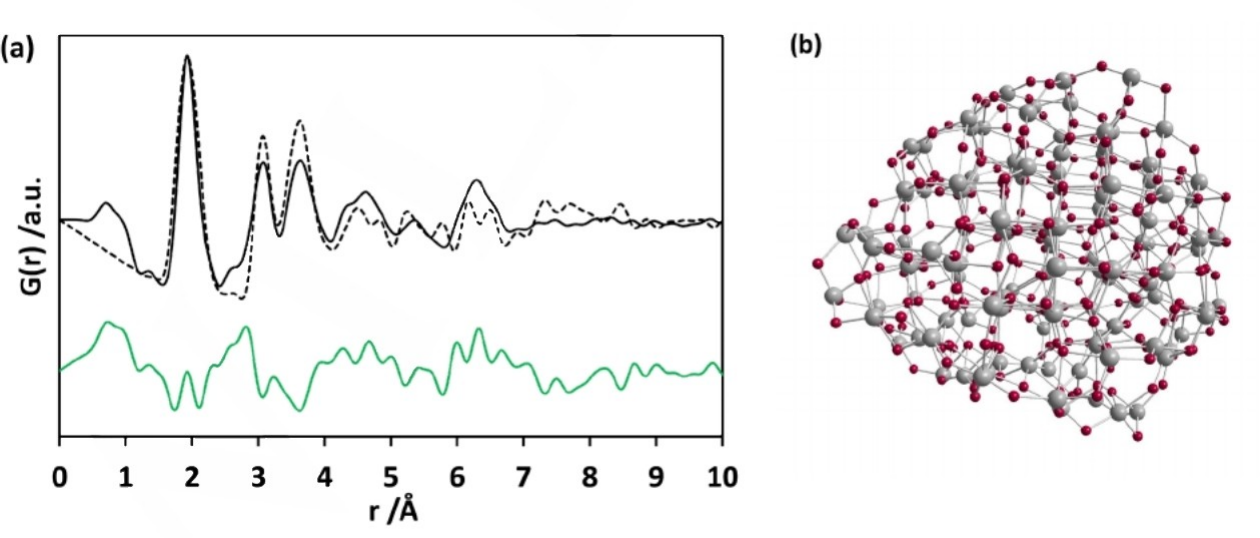
(a) PDF simulated for the model plotted as dashed line, compared to experimental PDF obtained from d‐Ti_x_O_y_ sample, while the difference (simulated PDF intensity subtracted from that of the experimental PDF) curve is given in green colour with an offset below. (b) Ti_123_O_246_ model particle given by Zhang et al.^[33]^

The pair correlation at 1.93 Å is typical for the Ti−O distance in TiO_6_ octahedra found in all TiO_2_ polymorphs. A significant rearrangement of the polyhedra has taken place during hydrolysis and most of the pentahedra from the precursor have been transformed into octahedra. The pair correlation at about 3 Å belongs to O−O distances. The experimental PDF of d‐Ti_x_O_y_ is damping at 7 Å (Figure 3a). Compared to the liquid Ti(OEt)_4_ precursor, which displays only weak pair correlations above 5 Å, the scattering clusters are slightly larger in the hydrolysed compound. After the elimination of the ethoxy ligands, more polyhedra are expected to connect and solid particles are rapidly formed in the suspension. The connection of several polyhedra leads to the formation of a wide variety of different titanium oxide clusters. These clusters were discussed in several studies.[^61^, ^62^, ^63^, ^64^, ^65^, ^66^] Some of them exhibit common structural motifs, composed of edge‐sharing TiO_6_ octahedra (Figure S5) forming M_3_O_13_ motifs.^[62]^ M_3_O_13_ structural units called triads are common building units found in polyoxometalates.^[67]^

For describing the structure of d‐Ti_x_O_y_, a model proposed by Zhang et al.^[33]^ fitted best with our data. The authors considered the effect of particle sizes on PDFs and carried out molecular‐dynamics (MD) simulations to generate input structures for RMC simulations of the atomic structure of amorphous titanium oxide nanoparticles. The structure model for a 2 nm‐sized TiO_2_ nanoparticle that best describes their experimental scattering data consists of a highly distorted shell and a small strained anatase‐like crystalline core (Figure 3b). The strained anatase structure of the particle with composition Ti_123_O_246_ comprises nearly two unit cells in one dimension.

Similar findings about the size of the ordered region were published by Fernández‐García et al.^[32]^ Ti−O atom pairs in the model show variations from the core to the shell region. The particle size of the model of 2 nm is close to the size estimated for our sample from gas adsorption measurements. Normally in the case of core‐shell particles, the bonds are either contracted or elongated, depending on the interior structure of the particle.^[68]^ The coordination number of the Ti atoms in the anatase core is six. The Ti−O distances get shorter heading from the core to the shell. Forces required to compensate distortions at the surface of the nanoparticles can also cause relatively longer bond lengths in the strained core. Consequently, the first neighbour Ti−O pair correlations populate around 1.94 Å (Figure S6). When the calculated PDF (Figure 3a) is compared to the experimental PDF of d‐Ti_x_O_y_, the theoretical value is only slightly larger than the Ti−O pair correlations observed for d‐Ti_x_O_y_ (1.93 Å). Both of these values remain slightly shorter in comparison to that in crystalline anatase (average out at 1.946 Å^[51]^).

To obtain information on the particle size, dynamic light scattering (DLS) data were collected from h‐Ti_x_O_y_. The data from the suspension after ultrasonication show a broad size distribution (Figure S7a) ranging from 20 nm to 3 μm. To maximise the contribution from the smaller particles, the suspension was left to sedimentation for 3 h. The sample then extracted from the middle portion of the suspension shows narrower particle size distribution (Figure S7b) ranging from 150 nm to 300 nm. While the experimental PDF points to structurally ordered regions smaller than 7 Å, DLS analysis reveals particles larger than 150 nm. This suggests that the particles are composed of small sub‐units with ordered cluster structures connected and surrounded by disordered regions as shown in Figure 3b. The particle size measured by DLS indicates the aggregation of such smaller entities. Creation of localised positive and negative charges originating from under‐coordinated O and Ti atoms at the surface of the nanoclusters is suggested as one of the reasons for the aggregation of titanium oxide particles.^[69]^ TEM images of d‐Ti_x_O_y_ display large aggregates with sizes ranging from tens to hundreds of nanometres (Figure S8). Based on the information obtained from DLS, TEM, and PDF analyses, h‐Ti_x_O_y_ consists of aggregates with a few hundred nanometres in size which contain ordered sub‐units of 7 Å in size. The specific surface area of d‐Ti_x_O_y_ was determined as 400 m^2^ ⋅ g^−1^ by nitrogen sorption measurements (for adsorption and desorption isotherms see Figure S9) using the non‐local density functional theory (NLDFT) method for data evaluation. Pore volume and pore diameter were calculated as 0.2 cm^3^ ⋅ g^−1^ and 2.6 nm.

### Local structure of h‐UV‐Ti_x_O_y_ and d‐UV‐Ti_x_O_y_

**Figure 4:**
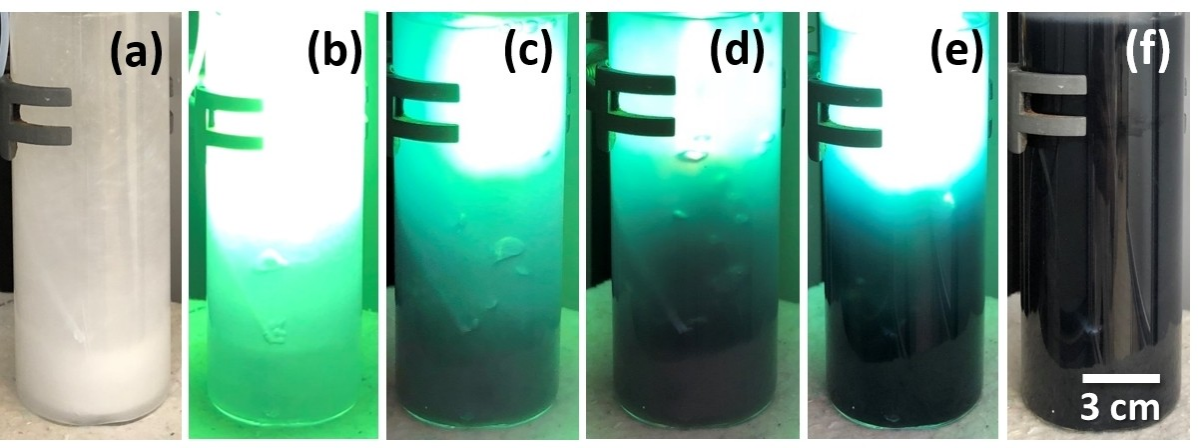
Photographs taken from the h‐UV‐Ti_x_O_y_ suspension. Following the injection of the Ti(OEt)_4_ precursor into the water‐methanol mixture: (a) before exposure to UV light, (f) when UV lamp is turned off after 2 h of exposure and (b–e) the states in between.

The non‐crystalline photocatalyst Ti_x_O_y_ changes colour under UV illumination. To track potential local structure changes by UV illumination, the samples inside the water‐methanol solution were exposed to UV light, providing the photocatalytic environment similar to previous studies investigating the H_2_ evolution activity of the materials.^[22]^ During synthesis, a white precipitate forms within the clear solution due to the hydrolysis of Ti(OEt)_4_ precursor injected into the water‐methanol mixture. The suspension was illuminated with UV light under an inert atmosphere. Several minutes of exposure induces a change in the colour of the suspension as shown in Figure 4. The suspension which is hazy white in the beginning gradually darkens and becomes dark blue/black after 15 min of UV light exposure. The rich colour attained persists until the UV illumination is turned off. The suspension at this condition necessitates handling under protective atmosphere and is named h‐UV‐Ti_x_O_y_. When exposed to air, O_2_ oxidizes the dark blue/black suspension, turning its colour back to white. Therefore, samples collected from the dark suspension have been kept under protective atmosphere until analysis. The counterpart of this suspension without any exposure to UV light (h‐Ti_x_O_y_) was used as the reference sample to capture potential structural changes induced by UV illumination.

**Figure 5:**
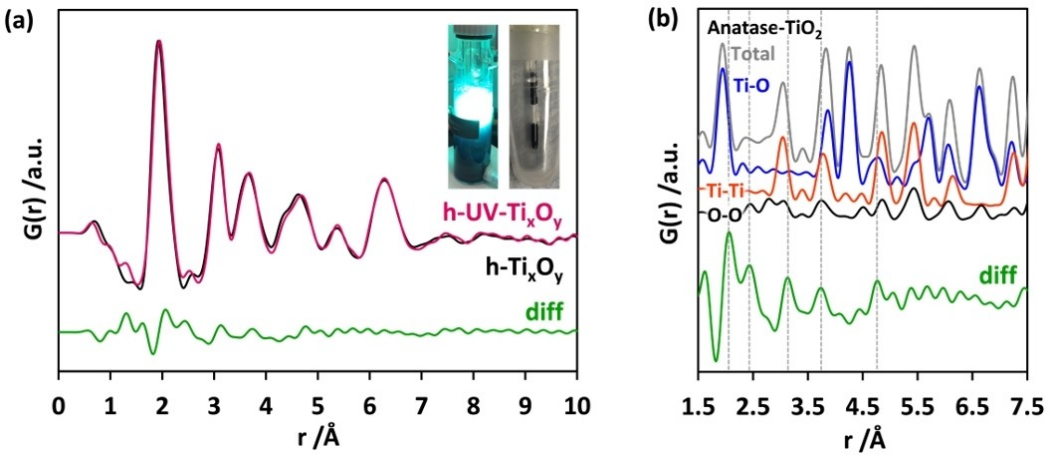
(a) Experimental PDFs obtained for h‐UV‐Ti_x_O_y_ (pink curve, photographs of the dark reaction suspension and the sealed capillary are given as inset) and h‐Ti_x_O_y_ (black curve) measured in reaction suspensions in sealed capillaries. The difference curve (diff, green coloured curve) is obtained by subtracting intensity of the PDF of h‐Ti_x_O_y_ from that of h‐UV‐Ti_x_O_y_. (b) Enlarged difference curve is compared to simulated PDFs for individual atom pairs in anatase. The dotted lines indicate the positions of the maxima of the difference curve.

The PDFs obtained for the two coloured suspensions h‐UV‐Ti_x_O_y_ (dark, filled into the capillary under protective atmosphere) and h‐Ti_x_O_y_ suspensions show only minor differences (Figure 5a). The difference curve obtained by subtracting the intensity of the PDF of h‐Ti_x_O_y_ from that of h‐UV‐Ti_x_O_y_ shows the existence of elongated Ti−O pairs in h‐UV‐Ti_x_O_y_ compared to h‐Ti_x_O_y_ (Figure 5b). Such an elongation of the interatomic bonds in the first Ti−O neighbourhood is possible for highly distorted Ti−O polyhedra. Upon UV light exposure, the colour change is regarded as evidence for Ti^3+^ formation^[70]^ triggering the creation of some other defects like oxygen vacancies in the vicinity.^[38]^ Ti−O bond lengths as long as 2.06 Å are reported on hydroxylated titanium oxide surfaces around Ti^3+^ defects.^[71]^

**Figure 6:**
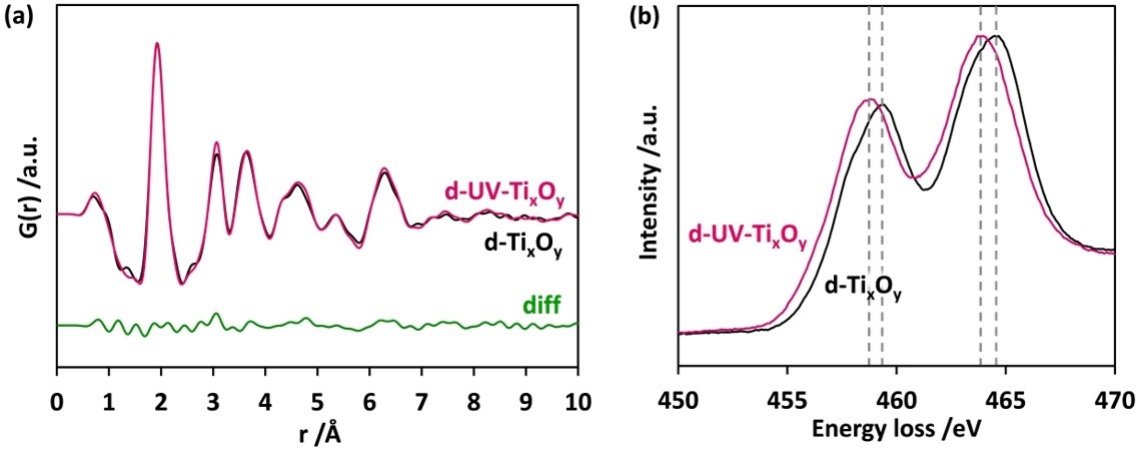
(a) Experimental PDFs obtained for d‐UV‐Ti_x_O_y_ (pink coloured curve) and d‐Ti_x_O_y_ (black coloured curve) from the data collected from two white powders. (b) EEL spectra of Ti L_2,3_ collected from d‐UV‐Ti_x_O_y_ compared to d‐Ti_x_O_y_ (normalised and averaged).

The powder obtained after drying the sediment of the suspension in air (d‐UV‐Ti_x_O_y_, white) has also been used for analysis. The counterpart of this powder was obtained from the suspension without any exposure to UV light (d‐Ti_x_O_y_) and used as the reference sample. The experimental PDF curves obtained for d‐UV‐Ti_x_O_y_ and d‐Ti_x_O_y_ after drying do not show significant differences (Figure 6a). The cluster sizes are in both cases slightly smaller than 7 Å.

X‐ray total scattering experiments and subsequent PDF analyses provide average structural information from the entire sample volume. The similarities between the two PDFs demonstrate that the structural changes due to irradiation are not significant regarding this technique. To have more insights into the potential changes caused by UV light exposure, d‐UV‐Ti_x_O_y_ and d‐Ti_x_O_y_ were examined by STEM and EELS (for the STEM images obtained from respective samples see Figure S10). EELS was carried out to obtain information on the presence of Ti^3+^ induced by UV illumination. Normalised and averaged EEL spectra of d‐UV‐Ti_x_O_y_ in comparison to d‐Ti_x_O_y_ are given in Figure 6b. Spectra collected from both d‐UV‐Ti_x_O_y_ and d‐Ti_x_O_y_ show two peaks of Ti L_2,3_ edges without further splitting, characteristic for an amorphous structure.^[72]^ Compared to the spectrum collected from d‐Ti_x_O_y_, the Ti L_2,3_ edge for d‐UV‐Ti_x_O_y_ shows a chemical shift of around 0.5 eV towards lower energy loss, suggesting the reduction of Ti^4+^ species to Ti^3+^.[^73^, ^74^] Even after exposing the samples to an oxygen‐rich atmosphere, traces of the reduced species remain. Multivariate statistical analysis^[75]^ of the EELS spectrum imaging (Figure S10) confirms that in the case of d‐UV‐Ti_x_O_y_ the particles are overall more reduced. Higher spatial resolution maps across the surface of the particles were not possible due to damage caused by electron beam irradiation.

### Heat‐induced crystallisation of d‐Ti_x_O_y_ to crystalline TiO_2_

**Figure 7:**
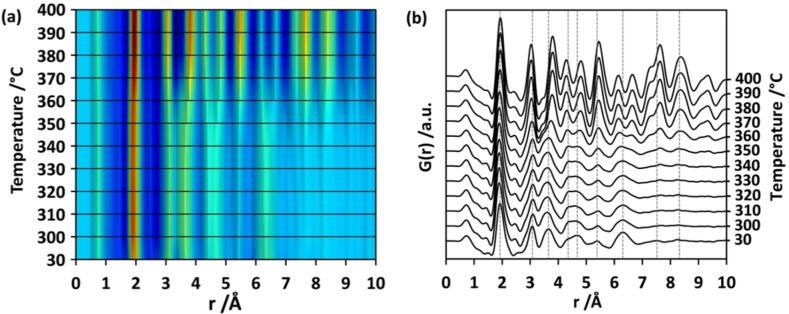
(a) PDFs obtained from in situ temperature‐dependent total scattering experiments of d‐Ti_x_O_y_. The temperature values at which the PDFs were obtained are represented on the y‐axis. Colour transition from blue to red indicates increase in the intensities of G(r). (b) Experimental PDFs obtained from in situ temperature‐dependent experiments given as a stack. Above 300 °C, the medium range order extends showing anatase‐like pair correlations.

To understand how the local structure of hydrolysed titanium ethoxide evolves during heat‐induced crystallisation, d‐Ti_x_O_y_ was studied in temperature‐dependent in situ X‐ray total scattering experiments. For this, d‐Ti_x_O_y_ was loaded into quartz glass capillaries with open ends and heated from 30 to 400 °C under a continuous flow of dry air. In a first experiment (respective PDF in Figure S11), data collection started at 30 °C and continued at temperatures from 50–350 °C with intervals of 50 °C. As major changes of the experimental PDFs are observed above 300 °C, the temperature range between 300–400 °C was examined in detail in a second experiment. PDFs were generated from the total scattering data measured every 10 °C. Changes of the intensities and positions of atom pairs are displayed by a colour map in Figure 7a, while the stack plot of the PDFs in Figure 7b shows peak splitting.

The experimental PDF obtained at 30 °C does not show pair correlations beyond 7 Å and the domains do not grow during the temperature increase up to 300 °C. The strained anatase‐like core seems to be maintained below 300 °C. Besides an expected broadening of the distribution of interatomic distances due to thermal motion is observed. Some of the peaks show a noticeable shape change with increasing temperature. The first Ti−O distance at 1.92 Å is a good example since it sharpens as the temperature increases, indicating that the distribution of Ti−O bond lengths gets narrower at higher temperatures. This points to successive ordering in the first Ti−O coordination. The change is accompanied by the appearance of a peak between 2.4–2.6 Å which is assigned to O−O pair distances as the partial PDFs calculated for anatase confirm (Figure S12). Starting at 30 °C, the pair correlation at 2.64 Å, belonging mainly to O−O pairs of edge‐sharing octahedra, first gains intensity and then shifts to 2.41 Å at 100 °C (Figure S11). This can be explained by the ordering of oxygen atoms towards an anatase‐like arrangement upon heating in air. Weak pair correlations above 7 Å start to gain intensity after heating to 340 °C. This is an indication of the formation of larger anatase‐like units. However, the low intensity of the pair correlations means that the number of larger units is still low compared to the small clusters. After heat treatment at 350 °C, these small peaks gain intensity and become visible (Figure S13). Additionally, several broad peaks start to split into distinct atom pairs with different intensities.

Next, the region in the experimental PDF associated with Ti−Ti distances appearing between 2.6 Å–3.3 Å (Figure 7b) displays changes. The first Ti−Ti peak at ∼3.1 Å, which represents the distance between titanium atoms of two edge‐sharing octahedra, does not significantly change position during heating. This indicates that in d‐Ti_x_O_y_ an anatase‐like Ti−Ti next nearest neighbourhood of edge‐sharing octahedra is already present after synthesis at 30 °C. Above 360 °C, this pair correlation gains intensity and gets narrower (Figure 7b). That means that successive structural ordering within and between the octahedra is taking place. The second Ti−Ti atom pair between two corner‐sharing octahedra at ∼3.68 Å shows a slight shift to longer interatomic distances of 3.78 Å at 400 °C. In addition, Ti−O atom pairs formed between neighbouring octahedra contribute to the pair correlation at 3.86 Å (Figure S12). Building defined Ti−O distances upon heating due to ordering of oxygen positions might be another reason for the observed peak shift in this region. Apart from changes observed in the close coordination spheres, alterations in the medium‐range become visible. In the interval 4.1 Å–5.1 Å, two broad peaks centred at 4.27 Å (Ti−O) and 4.85 Å (Ti−Ti) are observed at 340 °C. With increasing temperature, they sharpen and at 400 °C they are fitting perfectly with corresponding Ti−O and Ti−Ti distances of the anatase structure (Figure S12). The atom pair correlation appearing at larger distances also become well resolved as the temperature increases and can be assigned to defined distances in the anatase structure. The disordered parts in the strained anatase core become more and more ordered and at 400 °C, the experimental PDF fits best to anatase when compared also to the other titanium oxide polymorphs (Figure S14).

The refinement of the PDF obtained at 400 °C using the anatase crystal structure^[51]^ yields the calculated curve given in Figure S15 (for refinement parameters see Table S1). The results show that the anatase structure formed at 400 °C is still highly distorted. In particular, the calculated intensities for the peaks mainly representing the distance between Ti−Ti pairs of edge‐ and corner‐sharing octahedra (3.04 Å and 3.80 Å) and fourth nearest neighbour Ti−Ti pairs are lower than the observed intensities. The mismatch in these three peaks indicates a lower frequency of Ti−O pairs with respect to their frequency in a perfect anatase structure. Thus, a disorder of the O positions is present at 400 °C, which may point to the presence of oxygen defects. Despite the supply of oxygen during the experiments by the continuous air flow, these defects cannot be annihilated at this temperature within the given time frame. A similar observation was also reported by Mi et al.^[34]^ By constructing simple models with OH defects and correlating the defect concentration with the surface area, they identified the defects as OH defects at the surface of the particles where the structures are terminated. Fit to the experimental PDF obtained at 400 °C estimates a cluster size of 3.9 nm (Table S1) by coarsening of small clusters which were previously less than 1 nm in size. The sample obtained after the in situ experiments was later investigated by high‐resolution transmission electron microscopy (Figure S16). Small nanoparticles are embedded in a non‐crystalline, amorphous matrix.

However, the mechanism of coarsening, whether it occurs via Ostwald ripening where the larger grains/clusters grow at the expense of smaller ones, or via oriented attachment of these grains/clusters, which was previously hindered by surface groups, necessitates more detailed studies. The FTIR signal corresponding to adsorbed water on TiO_2_ ^[76]^ is present in the spectra collected from d‐Ti_x_O_y_ at room temperature (Figure S17), both before and after heating. Considering that d‐Ti_x_O_y_ contains an amorphous shell, it can be suggested that the oxygen defects present in d‐Ti_x_O_y_ before heating are predominantly OH defects present at the surface of the particles. Signals corresponding to organic surface groups were not observed both before and after crystallisation. In the thermogravimetric (TG) analysis (Figure S18), d‐Ti_x_O_y_ shows one weight loss step varying between 17–19 % until 510 °C which may include removal of alkoxide groups as well as water. Differential scanning calorimetry (DSC) curves (Figure S18) collected at different heating rates simultaneously with TG, show mainly exothermic peaks between 396–426 °C, which are attributed to crystallisation events. From the peak positions in the DSC curves obtained at different hating rates, the activation energy for a specific process can be calculated according to the method reported by Kissinger.^[77]^ The activation energy for the crystallisation process is calculated from the Kissinger plot as 195 kJ ⋅ mol^−1^ (Figure S18).

## Conclusion

The present study sheds light on the local structure of non‐crystalline titanium oxide (Ti_x_O_y_) photocatalysts synthesised from a titanium (IV) ethoxide [Ti(OEt)_4_] precursor. By pair distribution function (PDF) analysis, the liquid Ti(OEt)_4_ precursor is confirmed to form trimers consisting of corner‐sharing Ti−O pentahedra. During hydrolysis, the trimeric structure grows by rearrangement of the primary building units and the condensation of Ti−O polyhedra. Experimental PDFs reveal the presence of ordered domains represented by an anatase‐like core of 7 Å in size surrounded by an amorphous disordered shell. During illumination with UV light, the h‐Ti_x_O_x_ suspension turns from milky white to dark blue/black. The experimental PDFs show a slight elongation of the Ti−O bonds during UV treatment. The change of the Ti−O coordination is caused by the reduction of Ti^4+^ to Ti^3+^ species, which is accompanied by the formation of oxygen defects. The dry powders with and without UV illumination were examined by EELS. A shift in the Ti L_2,3_ edge indicating the reduction to Ti^3+^ was observed for the UV illuminated and dried powder. Finally, crystallisation was monitored during in situ temperature‐dependent total scattering experiments. Significant crystal growth of the anatase polymorph appears above 350 °C. Refinements of the PDF models to the data collected at 400 °C point to the presence of oxygen defects inherited from the amorphous structure. The present study is a contribution to the identification of common motifs in the local structure of amorphous titanium oxides and the crystalline counterparts.

## Conflict of interest

The authors declare no conflict of interest.

## Supporting information

As a service to our authors and readers, this journal provides supporting information supplied by the authors. Such materials are peer reviewed and may be re‐organized for online delivery, but are not copy‐edited or typeset. Technical support issues arising from supporting information (other than missing files) should be addressed to the authors.

Supporting Information

## References

[1] A. Fujishima, K. Honda, Nature 1972, 238, 37–38. 12635268 10.1038/238037a0

[2] A. Fujishima, X. T. Zhang, D. A. Tryk, Surf. Sci. Rep. 2008, 63, 515–582.

[3] D. Zhang, S. Dong, Prog. Nat. Sci. Mater. Int. 2019, 29, 277–284.

[4] T. J. Miao, J. Tang, J. Chem. Phys. 2020, 152, 194201. 33687236 10.1063/5.0008537

[5] X. Pan, M.-Q. Yang, X. Fu, N. Zhang, Y.-J. Xu, Nanoscale 2013, 5, 3601–3614. 23532413 10.1039/c3nr00476g

[6] J. Liu, Z. Wei, W. Shangguan, ChemCatChem 2019, 11, 6177–6189.

[7] S. G. Kumar, L. G. Devi, J. Phys. Chem. A 2011, 115, 13211–13241. 21919459 10.1021/jp204364a

[8] M. Anpo, M. J. Takeuchi, J. Catal. 2003, 216, 505–516.

[9] I. Nakamura, N. Negishi, S. Kutsuna, T. Ihara, S. Sugihara, K. Takeuchi J. Mol. Catal. A 2000, 161, 205–212.

[10] A. Weir, P. Westerhoff, L. Fabricius, N. von Goetz, Environ. Sci. Technol. 2012, 46, 2242–2250. 22260395 10.1021/es204168d PMC3288463

[11] D. A. H. Hanaor, C. C. Sorrell, J. Mater. Sci. 2011, 46, 855–874.

[12] H. Zhang, J. F. Banfield, J. Mater. Chem. 1998, 8, 2073–2076.

[13] H. Zhang, J. F. Banfield, J. Phys. Chem. B 2000, 104, 3481–3487.

[14] C. T. Carneiro, T. J. Savenije, J. A. Moulijn, G. Mul, J. Phys. Chem. C 2010, 114, 327–332.

[15] Y. Kakuma, A. Y. Nosaka, Y. Nosaka, Phys. Chem. Chem. Phys. 2015, 17, 1–8. 10.1039/c5cp02004b 26120611

[16] W. Kim, T. Tachikawa, G.-H. Moon, T. Majima, W. Choi, Angew. Chem. Int. Ed. 2014, 53, 14036–14041, 10.1002/anie.201406625 25314627

[17] D. C. Hurum, A. G. Agrios, K. A. Gray, T. Rajh, M. C. Thurnauer, J. Phys. Chem. B 2003, 107, 4545–4549.

[18] T. Ohno, K. Sarukawa, K. Tokieda, M. Matsumura, J. Catal. 2001, 203, 82–86.

[19] B. Ohtani, O. O. Prieto-Mahaney, D. Li, R. Abe, J. Photochem. Photobiol. A 2010, 216, 179–182.

[20] J. Soria, J. Sanz, M. J. Torralvo, I. Sobrados, C. Garlisi, G. Palmisano, S. Çetinkaya, S. Yurdakal, V. Augugliaro, Appl. Catal. B 2017, 210, 306–319.

[21] M. J. Torralvo, J. Sanz, I. Sobrados, J. Soria, C. Garlisi, G. Palmisano, S. Çetinkaya, S. Yurdakal, V. Augugliaro, Appl. Catal. B 2018, 221, 140–151.

[22] D. Zywitzki, H. Jing, H. Tüysüz, C. K. Chan, J. Mater. Chem. A 2017, 5, 10957–10967.

[23] M. Benmami, K. Chhor, A. V. Kanaevt, J. Phys. Chem. B 2005, 109, 19766–19771. 16853556 10.1021/jp051396+

[24] Z. Zhang, P. A. Maggard, J. Photochem. Photobiol. A 2007, 186, 8–13.

[25] J. Li, S. Liu, Y. He, J. Wang, Microporous Mesoporous Mater. 2008, 115, 416–425.

[26] Y. Li, T. Sasaki, Y. Shimizu, N. Koshizaki, J. Am. Chem. Soc. 2008, 130, 14755–14762. 18844352 10.1021/ja805077q

[27] C. Randorn, J. T. S. Irvine, P. Robertson, Int. J. Photoenergy 2008, 426872, 1–6.

[28] S. Buddee, S. Wongnawa, U. Sirimahachai, W. Puetpaibool, Mater. Chem. Phys. 2011, 126, 167–177.

[29] T. Grewe, H. Tüysüz, ChemSusChem 2015, 8, 3084–3091. 26261010 10.1002/cssc.201500774

[30] V. Petkov, G. Holzhüter, U. Tröge, T. Gerber, B. Himmel J. Non-Cryst. Solids 1998, 231, 17–30.

[31] M. Gateshki, S. Yin, Y. Ren, V. Petkov, Chem. Mater. 2007, 19, 2512–2518.

[32] M. Fernández-García, C. Belver, J. C. Hanson, X. Wang, J. A. Rodriguez, J. Am. Chem. Soc. 2007, 129, 13604–13612. 17927180 10.1021/ja074064m

[33] H. Zhang, B. Chen, J. F. Banfield, Phys. Rev. B 2008, 78, 214106.

[34] J. L. Mi, K. M. Ø. Jensen, C. Tyrsted, M. Bremholm, B. B. Iversen, CrystEngComm 2015, 17, 6868–6877.

[35] J. Ma, K. G. Reeves, A.-G. P. Gutierrez, M. Body, C. Legein, K. Kakinuma, O. J. Borkiewicz, K. W. Chapman, H. Groult, M. Salanne, D. Dambournet, Chem. Mater. 2017, 29, 8313–8324.

[36] M. Anpo, M. Che, B. Fubini, E. Garrone, E. Giamello, M. C. Paganini, Top. Catal. 1999, 8, 189–198.

[37] C. Di Valentin, G. Pacchioni, J. Phys. Chem. C 2009, 113, 20543–20552.

[38] L.-B. Xiong, J.-L. Li, B. Yang, Y. Yu, J. Nanomater. 2012, 831524, 1–13.

[39] L. Zhang, B. K. Miller, P. A. Crozier, Nano Lett. 2013, 13, 679–684. 23294377 10.1021/nl304333h

[40] M. Kus, T. Altantzis, S. Vercauteren, I. Caretti, O. Leenaerts, K. J. Batenburg, M. Mertens, V. Meynen, B. Partoens, S. Van Doorslaer, S. Bals, P. Cool, J. Phys. Chem. C 2017, 121, 26275–26286.

[41] S. Jayashree, M. Ashokkumar, Catalysts 2018, 8, 1–26.

[42] M. Qiu, Y. Tian, Z. Chen, Z. Yang, W. Li, K. Wang, L. Wang, K. Wang, W. Zhang, RSC Adv. 2016, 6, 74376–74383.

[43] R. F. Howe, M. Gratzel, J. Phys. Chem. 1985, 89, 4495–4499.

[44] M. Graetzel, R. F. Howe, J. Phys. Chem. 1990, 94, 2566–2572.

[45] T. Berger, M. Sterrer, O. Diwald, E. Knözinger, D. Panayotov, T. L. Thompson, J. T. Yates, J. Phys. Chem. B 2005, 109, 6061–6068. 16851666 10.1021/jp0404293

[46] K. Komaguchi, H. Nakano, A. Araki, Y. Harima, Chem. Phys. Lett. 2006, 428, 338–342.

[47] R. Khanam, D. Taparia, B. Mondal, D. Mohanta, Appl. Phys. A 2016, 122, 1–7.

[48] X. Chen, L. Liu, P. Y. Yu, S. S. Mao, Science 2011, 331, 746–750. 21252313 10.1126/science.1200448

[49] A. Folger, P. Ebbinghaus, A. Erbe, C. Scheu, ACS Appl. Mater. Interfaces 2017, 9, 13471–13479. 28355873 10.1021/acsami.7b01160

[50] J. Livage, M. Henry, C. Sanchez, Prog. Solid State Chem. 1988, 18, 259–341.

[51] M. Horn, C. Schwebdtfeger, E. Meagher, Z. Kristallogr. Cryst. Mater. 1972, 136, 273–281.

[52] I. S. Ignatyev, M. Montejo, J. J. López González, J. Phys. Chem. A 2007, 111, 7973–7979. 17636972 10.1021/jp073423x

[53] J. A. Ibers, Nature 1963, 197, 686–687.

[54] D. A. Wright, D. A. Williams, Acta Crystallogr. 1968, B24, 1107–1114.

[55] W. R. Russo, W. H. Nelson, J. Am. Chem. Soc. 1970, 92, 1521–1526.

[56] F. Babonneau, S. Doeuff, A. Leaustic, C. Sanchez, C. Cartier, M. Verdaguer, Inorg. Chem. 1988, 27, 3166–3172.

[57] R. L. Martin, G. Winter, J. Chem. Soc. 1961, 2947–2957.

[58] C. N. Caughlan, H. S. Smith, W. Katz, W. Hodgson, R. W. Crowe, J. Am. Chem. Soc. 1951, 73, 5652–5654.

[59] H. Weingarten, J. R. Van Wazer, J. Am. Chem. Soc. 1965, 87, 724–730.

[60] S. Weymann-Schildknetch, M. Henry, J. Chem. Soc. Dalton Trans. 2001, 2425–2428.

[61] K. Watenpaugh, C. N. Caughlan, Chem. Commun. 1967, 2, 76–77.

[62] R. Schmid, A. Mosset, J. Galy, J. Chem. Soc. Dalton Trans. 1991, 1999–2005.

[63] Y. Chen, E. Trzop, A. Makal, J. D. Sokolow, P. Coppens, Inorg. Chem. 2013, 52, 4750–4752. 23587091 10.1021/ic302692d

[64] O. Lamiel-Garcia, A. Cuko, M. Calatayud, F. Illas, S. T. Bromley, Nanoscale 2017, 9, 1049–1058. 27809322 10.1039/c6nr05788h

[65] Á. Morales-García, A. M. Escatllar, F. Illas, S. T. Bromley, Nanoscale 2019, 11, 9032–9041. 31021336 10.1039/c9nr00812h

[66] Z.-W. Qu, G.-J. Kroes, J. Phys. Chem. B 2006, 110, 8998–9007. 16671707 10.1021/jp056607p

[67] A. Kondinski, T. N. Parac-Vogt, Front. Chem. 2018, 6, 1–7. 30159307 10.3389/fchem.2018.00346 PMC6104420

[68] W. J. Huang, R. Sun, J. Tao, L. D. Menard, R. G. Nuzzo, J. M. Zuo, Nat. Mater. 2008, 7, 308–313. 18327263 10.1038/nmat2132

[69] M. Alimohammadi, K. A. Fichthorn, Nano Lett. 2009, 9, 4198–4203. 19719155 10.1021/nl9024215

[70] V. M. Khomenko, K. Langer, H. Rager, A. Fett, Phys. Chem. Miner. 1998, 25, 338–346.

[71] X.-X. Zou, G.-D. Li, K.-X. Wang, L. Li, J. Su, J.-S. Chen, Chem. Commun. 2010, 46, 2112–2114. 10.1039/b924840d 20221509

[72] G. Bertoni, E. Beyers, J. Verbeeck, M. Mertens, P. Cool, E. F. Vansant, G. Van Tendeloo, Ultramicroscopy 2006, 106, 630–635.

[73] N. L. De Silva, A. C. A. Jayasundera, A. Folger, O. Kasian, S. Zhang, C.-F. Yan, C. Scheu, J. Bandara, Catal. Sci. Technol. 2018, 8, 4657–4664.

[74] E. Stoyanov, F. Langenhorst, G. Steinle-Neumann, Am. Mineral. 2007, 92, 577–586.

[75] S. Zhang, C. Scheu, Microscopy 2018, 67, i133–i141. 29136225 10.1093/jmicro/dfx091 PMC7207561

[76] H. Belhadj, A. Hakki, P. K. J. Robertson, D. W. Bahnemann, Phys. Chem. Chem. Phys. 2015, 17, 22940–22946. 26266701 10.1039/c5cp03947a

[77] H. E. Kissinger, J. Res. Natl. Bur. Stand. 1956, 57, 217–221.

