# Supplementary material for: Monitoring the Structure Evolution of Titanium Oxide Photocatalysts: From the Molecular Form via the Amorphous State to the Crystalline Phase
Source: Chemistry. 2021 Jul 9;27(45):11600–8. doi: 10.1002/chem.202101117 (PMC8456846; doi:10.1002/chem.202101117)
Supplement: Supplementary file 1 — Supporting Information [file CHEM-27-11600-s001.pdf]

Chemistry–A European Journal

Supporting Information

Monitoring the Structure Evolution of Titanium Oxide Photocatalysts: From the Molecular Form via the Amorphous State to the Crystalline Phase

Ezgi Onur Şahin, Yitao Dai, Candace K. Chan, Harun Tüysüz, Wolfgang Schmidt, Joohyun Lim, Siyuan Zhang, Christina Scheu, and Claudia Weidenthaler*

Contents

Experimental section

Synthesis procedure……2

Experimental parameters for the total scattering experiments……2

Characterisation……3

Figure S1 XRD pattern collected from d-Ti_xO_y……4

Figure S2 PDFs obtained after synthesis using three different titanium alkoxides……4

Figure S3 PDFs obtained from liquid $Ti(OEt)_4$ and h-Ti_xO_y……5

Figure S4 PDFs obtained from h-Ti_xO_y and d-Ti_xO_y……5

Figure S5 Structure of crystallised $Ti_4O_{16}C_{16}$ and PDF simulated using this structure……6

Figure S6 Components of the simulated PDF for $Ti_{123}O_{246}$ particle……6

Figure S7 DLS results obtained from h-Ti_xO_y……7

Figure S8 TEM images obtained from d-Ti_xO_y ……7

Figure S9 Nitrogen adsorption/desorption isotherms of d-Ti_xO_y……8

Figure S10 STEM images and EELS spectrum images acquired from d-UV-Ti_xO_y and d-Ti_xO_y……9

Figure S11 Stack plot of PDFs obtained from *in situ* total scattering data collected from d-Ti_xO_y……10

Figure S12 Partial PDFs of the atom pairs calculated from anatase-TiO_2 structure……10

Figure S13 Scattering data and respective long range PDFs……11

Figure S14 PDFs simulated using anatase, rutile and brookite……11

Figure S15 Refinement of the PDF obtained from d-Ti_xO_y at 400 °C using anatase structure……12

Table S1 Refined parameters for the PDF obtained from d-Ti_xO_y at 400 °C using anatase-TiO_2 structure……12

Figure S16 High-resolution transmission electron micrographs obtained after *in situ* temperature dependent total scattering experiments……13

Figure S17 FTIR spectra obtained from d-Ti_xO_y and d-Ti_xO_y after heating to 400 °C……14

Figure S18 DSC and TG data collected for d-Ti_xO_y and respective Kissinger plot……14

References……15

Experimental section

Synthesis procedure

The adopted synthesis method,[1] so called 'direct injection', is a simple synthesis procedure to obtain high surface area titanium oxide (Ti_xO_y) photocatalysts involving simultaneous test for photocatalytic water splitting. The method involves direct injection of titanium(IV) ethoxide [$Ti(OEt)_4$] precursor into the water-methanol reaction mixture in a photocatalytic reaction cell equipped with a lamp to provide radiation in ultraviolet (UV) range. With this method, synthesised catalyst is exposed to UV light for 2 h mimicking direct injection synthesis method. The synthesis product in suspension is referred to as h-UV-Ti_xO_y. To have a non-UV counterpart, samples were also synthesised via '*ex situ'* synthesis method, this time in the absence of UV light. The non-UV synthesis product in suspension is referred to as h-Ti_xO_y. The two synthesis routes are explained below in detail. The samples resulting from both of following synthesis routes were collected for stationary suspension measurements in sealed capillaries. Protective atmosphere was maintained in the case of air sensitive (suspension subjected to UV irradiation) samples. In order to have dry powder samples, the sediments were separated from the suspensions by decanting off the liquid part. The remaining parts were dried overnight (for 3 h in case of powders obtained as counterparts to suspensions measured at the synchrotron source due to limited time) at 60 °C in an oven to obtain d-UV-Ti_xO_y and d-Ti_xO_y samples.

Synthesis procedure under UV light exposure (h-UV-Ti_xO_y): Synthesis of h-UV-Ti_xO_y samples involves injection of 525 µL $Ti(OEt)_4$ (99.98%, Sigma Aldrich, CAS: 3087-36-3) as metal oxide precursor into a mixture of 180 mL deionized water and 20 mL MeOH (Sigma Aldrich, CAS: 67-56-1) in a glass photocatalytic reaction vessel. This concentration was calculated aiming 1 $g\cdot L^{-1}$ TiO_2 catalyst in the end. The reaction vessel is equipped with a Peschl Ultraviolet TQ150 150 W middle pressure Hg-lamp having peak emission at 366 nm for UV light exposure. The UV lamp is inserted to the reaction vessel with a water cooled jacket. Following the injection of the precursor into the reaction solution, the UV lamp is turned on. During illumination, the reaction suspension was stirred using a stirring bar and kept under continuous Ar purging (50 $mL\cdot min^{-1}$). To be consistent with the previous photocatalytic study, the irradiation period was kept at 2 h.

Synthesis procedure without UV light exposure (h-Ti_xO_y): To observe the effects of UV light exposure, the synthesis was also carried out in the absence of UV light. The same amount of the precursor was injected into the reaction mixture prepared in the same way. In this case, samples were synthesised in a beaker rather than the reaction vessel. The reaction mixture was stirred for 2 h under continuous Ar purging. In this way h-Ti_xO_y 'non-UV' samples were obtained which are interesting for us to understand the nature of the amorphous Ti_xO_y material independent of the effects induced by UV light exposure. These samples together with the dried version, d-Ti_xO_y, are studied extensively including the *in situ* temperature-dependent experiments in the present work.

Experimental parameters for the total scattering experiments

Room temperature PDFs: Data collection for the powder samples, suspensions and the liquid precursor were performed at I15-1 beamline at Diamond Light Source (Diamond). The energy was 77 keV (λ= 0.16167 Å), the sample to detector distance was 200 mm (Q_{min} = 0.2 $Å^{-1}$ and $Q_{max,\ instrumental}$ = 38 $Å^{-1}$), and the beam size on the sample was 700 x 150 µm. Data acquisition was performed with a Perkin Elmer XRD 4343 CT detector. Collected scattering data were integrated using the DAWN software package.[2] Scattering data obtained from an empty capillary and a capillary filled with the water- methanol mixture were used as backgrounds for powder and suspension measurements. The program PDFgetX3[3] implemented in the xPDFsuite[4] was used for processing PDFs from the integrated scattering data (q_{max}= 22 $Å^{-1}$). DebyePDFCalculator within Diffpy-CMI[5] (q_{max}= 22 $Å^{-1}$, ADPs= 0.003, delta2= 1.0, Q_{damp}= 0.0258 $Å^{-1}$, Q_{broad}= 0.0118 $Å^{-1}$) was used to simulate PDFs for non-periodic models.

Room temperature PDF measurements for samples d-UV-Ti_xO_y (24 h UV exposure) and d-Ti_xO_y were carried out in-house using STOE Stadi-P diffractometer in transmission mode using Mo-Kα radiation (λ= 0.7093 Å) equipped with a primary Ge(111) monochromator and a position sensitive Mythen1K detector. The data collection range was 5-120° 2θ with a step width of 0.015° 2θ using variable counting time. Collected data were transformed into PDFs (q_{min}= 0.1 $Å^{-1}$ and q_{max}= 15 $Å^{-1}$).

In situ temperature-dependent PDF experiments: In situ temperature-dependent experiments were performed on d-Ti_xO_y at beamline P02.1 at PETRA III, Deutsches Elektronen-Synchrotron (DESY) [energy: 60 keV (λ= 0.20723 Å); detector: Perkin Elmer XRD 1621; sample to detector distance: 250.88 mm; Q_{min}= 0.4 $Å^{-1}$ and $Q_{max,\ instrumental}$= 34.2 $Å^{-1}$, beam size: 1 x 1 mm]. Powder samples were packed in both ends open quartz capillaries (length 100 mm, inner diameter 0.9 mm, wall thickness 0.15 mm) ensuring a few centimetres of sample length. The capillaries mounted on the custom-made cell were heated at a rate of 10 $K\cdot min^{-1}$ using a hot air blower. The temperature was calibrated based on the trend obtained from the reading of the thermocouple inserted inside the empty quartz capillary heated in the same way as the samples. The temperature calibration curve and temperature vs. time curve are given below:

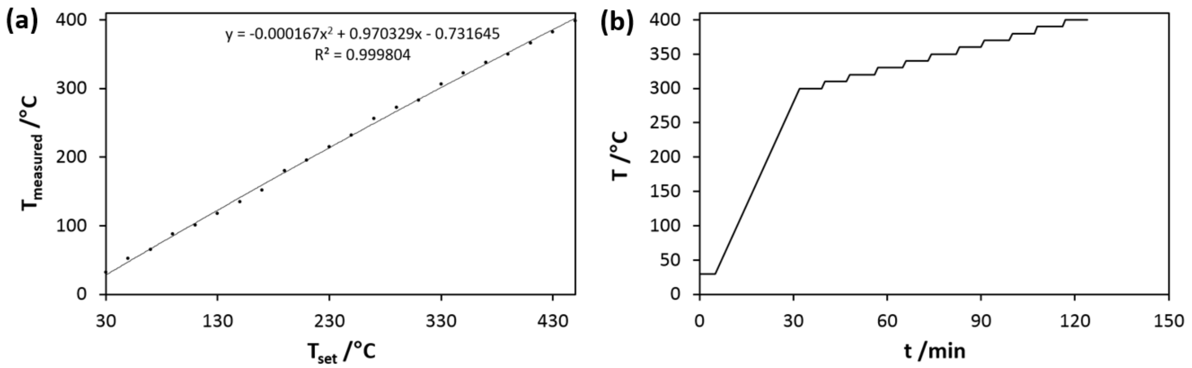

(a) Temperature calibration curve used for setting the temperature for the hot air blower and (b) temperature vs. time curve for the 30 °C- 400 °C *in situ* temperature-dependent experiments. Data were taken for 5 min starting from 30 °C at each 10 °C in the 300-400 °C interval where the heating rate was kept constant at 10 °C·min^{-1}.

During heating, the powder was kept under synthetic air with a flow of 5 mL·min^{-1}. Data were collected in temperature steps of 10 °C between 300 and 400 °C. The data collection time per frame was 5 min. The DAWN software package was used to integrate scattering data. PDFs were generated (q_{max}= 22 Å^{-1}) using PDFgetX3 implemented in the xPDFsuite, simulated (q_{max}= 22 Å^{-1}, ADPs= 0.003, delta2=1.0, Q_{damp}= 0.0375 Å^{-1}, Q_{broad}= 4.067e-06 Å^{-1}) and was fitted using PDFgui.[6] The false colour heat map was obtained using xPDFsuite.

Characterisation

TEM imaging was performed using a Titan Themis instrument (Thermo Fisher Scientific) operated at 300 kV with a Cs corrector for the image forming lens. EELS data were acquired in a probe corrected Titan Themis instrument (Thermo Fisher Scientific) operated at 300 kV using a Gatan Quantum ERS energy filter with the entrance aperture collecting electrons up to 35 mrad. Multivariate statistical analysis was performed on the datasets to highlight the different spatial contributions of the Ti $L_{2,3}$ edges.[7] High resolution TEM images shown in the Supporting Information were taken with a Hitachi HF-2000 instrument. Specific surface area was determined by nitrogen sorption experiments with a Quantachrome NOVA 3200e instrument after degassing approximately 150 mg powder samples at 150 °C overnight. Data were evaluated by the BET (Brunauer-Emmett-Teller) method using the adsorption data in the relative pressure range of 0.05 to 0.2. From the specific surface area, approximate particle sizes can be calculated using the relation D_p= 6000·σ^{-1}·A_s^{-1} (assuming spherical particles, D_p = particle size in nm, σ = specific density in g·cm^{-3}, A_s = specific surface area in m^2·g^{-1}). Dynamic light scattering (DLS) data were recorded on a Malvern Zetasizer Nano-ZS using laser radiation with a wavelength of 633 nm. The scattered light was measured at a backscattering angle of 173°. The suspension collected after synthesis was ultrasonicated for 30 min. The results of this collection was compared to the ones collected from the middle portion of the suspension let settle for 3 h. Thermogravimetry (TG) and differential scanning calorimetry (DSC) measurements were carried out using a NETZSCH STA 449 F3 Jupiter thermal analyser for the qualitative analysis of crystallisation temperatures. The measurements were carried out under an air flow of 40 mL·min^{-1} using approximately 10 mg powder heated in an aluminium oxide crucible with heating rates of 2, 5 and 10 °C·min^{-1}. Fourier transform infrared (FTIR) spectroscopy measurements were performed using a Perkin Elmer Spectrum Two spectrometer with an attenuated total reflectance (ATR) unit. For construction of the clusters Avogadro molecular editor[8] and for visualisation of the structures Diamond software[9] is used.

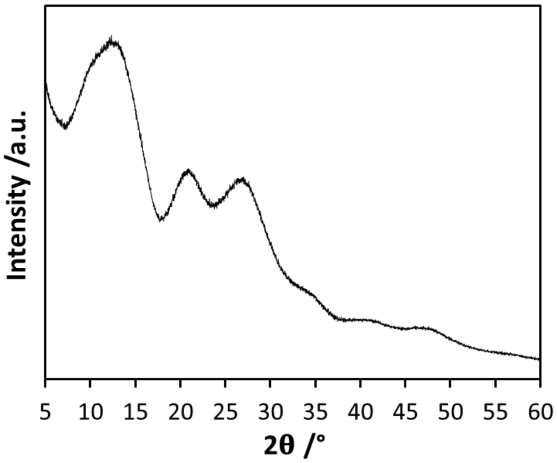

Figure S1 X-ray diffraction (XRD) pattern collected from d-Ti_xO_y using in-house instrument (λ= 0.7093 Å).

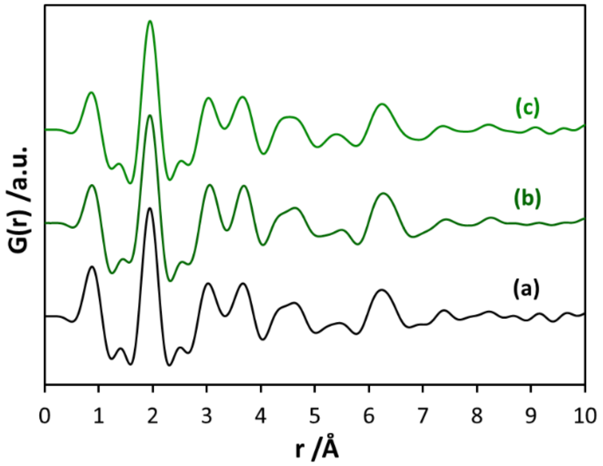

Figure S2 PDFs obtained from total scattering experiments performed with dried powders obtained after synthesis using three different titanium alkoxides: (a) $Ti(OEt)_4$, (b) $Ti(OBu)_4$ and (c) $Ti(OiPr)_4$. Total scattering data were collected at the European Synchrotron Radiation Facility (ESRF). Note that the PDFs show an unreal feature below 1 Å due to Fourier transformation of scattering data obtained at limited maximum momentum transfer (q_{max}= 14 $Å^{-1}$).

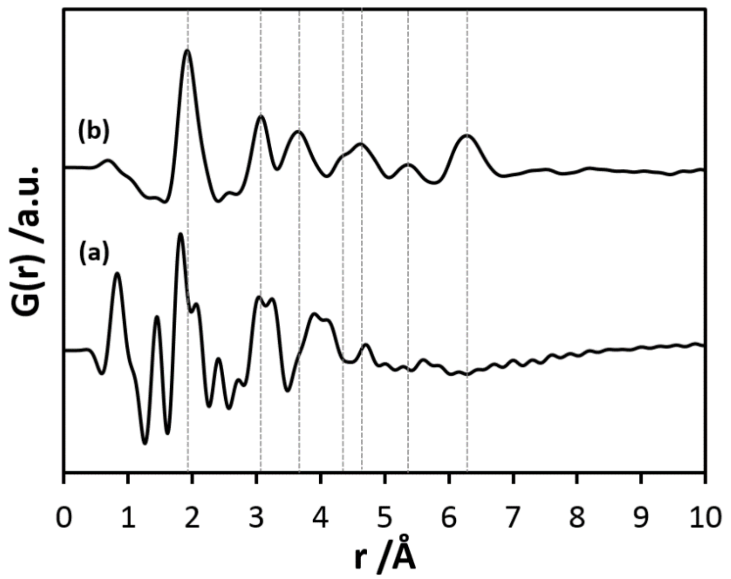

Figure S3 PDFs obtained from (a) liquid $Ti(OEt)_4$ precursor measured in a capillary sealed under protective atmosphere and (b) h-Ti_xO_y (sample in suspension).

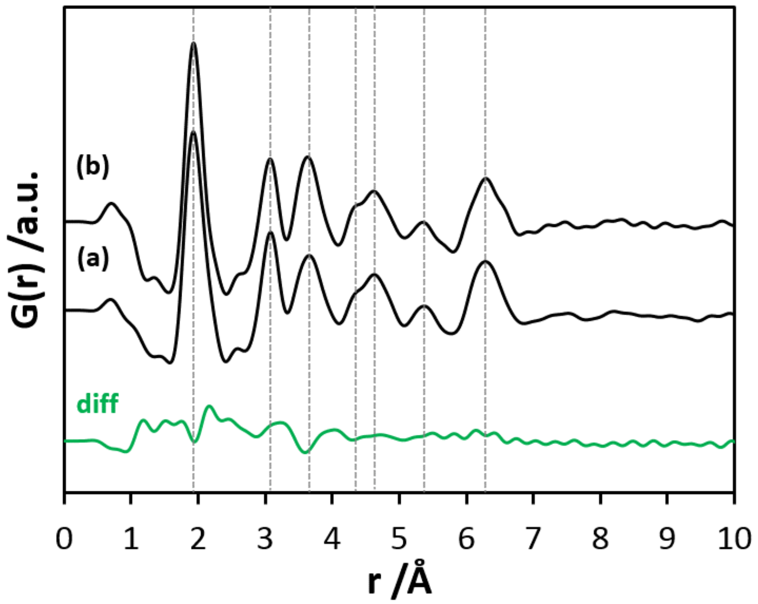

Figure S4 PDFs obtained from (a) h-Ti_xO_y (sample in suspension) and (b) d-Ti_xO_y (dried powder) samples. Note that the intensities are normalised according to the most intense peak. Difference curve (green curve, intensities of b, subtracted from a) is plotted below for easy comparison.

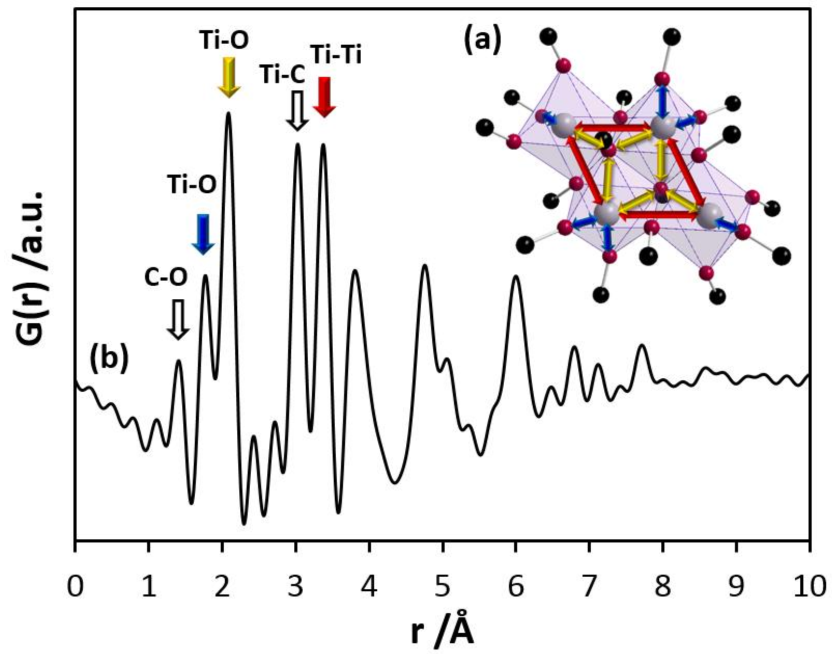

Figure S5 (a) Structure of crystallised $Ti_4O_{16}C_{16}$ in solid state given by Wright and Williams[10] and (b) simulated PDF.

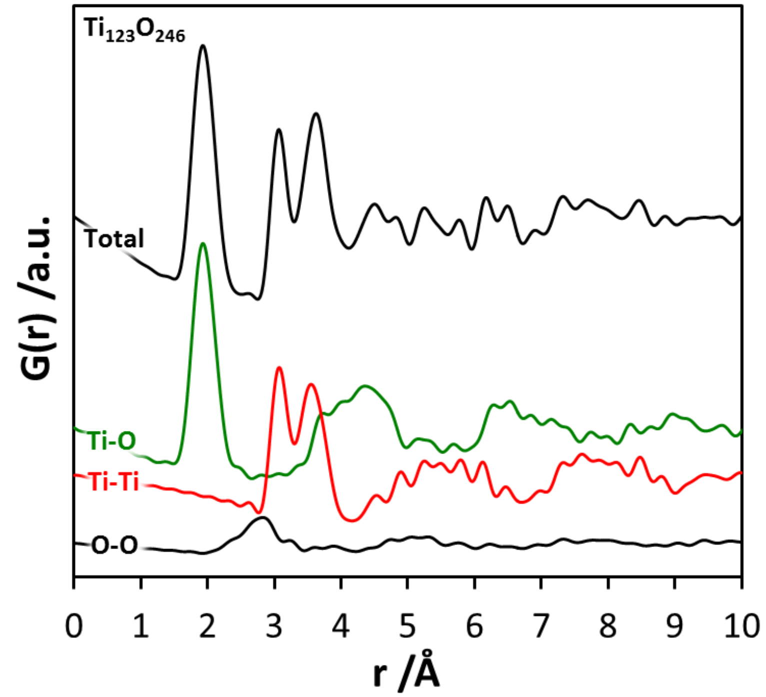

Figure S6 Components of the simulated PDF for $Ti_{123}O_{246}$ particle.[11]

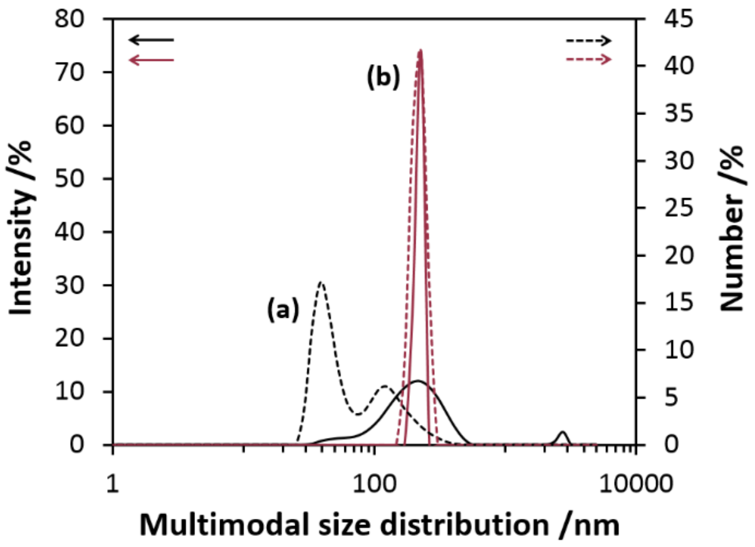

Figure S7 Particle size distribution based on the results of the dynamic light scattering obtained from h-Ti_xO_y after ultrasonication (a) representative of the whole suspension and (b) sample collected from the middle portion of the suspension left to sediment for 3 h. Solid lines show intensity percentages while dashed lines show number percentages of the particles. Note that the size values are plotted on logarithmic scale.

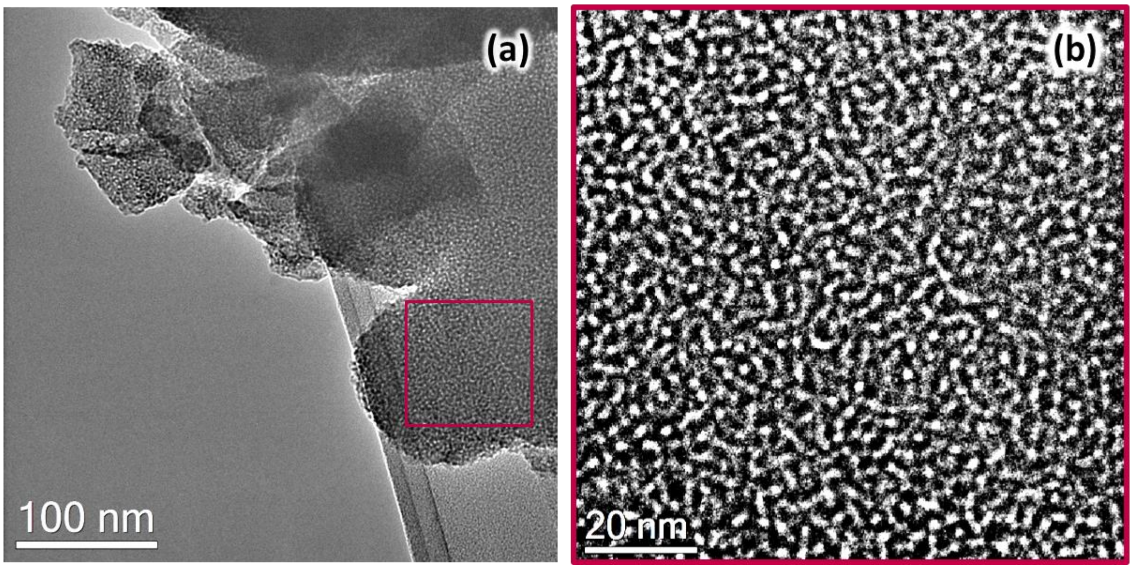

Figure S8 TEM images obtained from d-Ti_xO_y showing (a) aggregates and (b) presence of short-range order in the enlarged area marked by a red rectangle in (a).

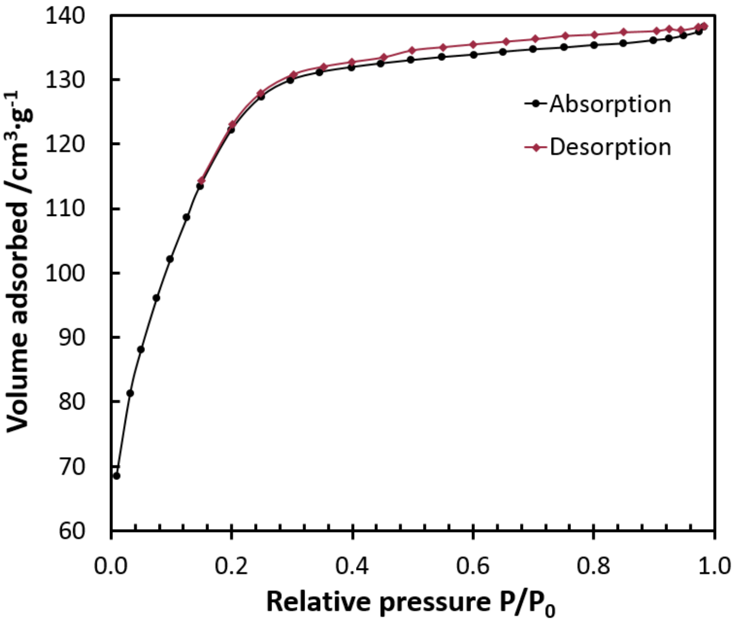

Figure S9 Nitrogen adsorption/desorption isotherms of d-Ti_xO_y sample after degassing approximately 160 mg powder at 150 °C overnight (15.9 wt% loss). The isotherm is of type Ib according to the IUPAC definition. Separation of the processes of supermicropore filling and monolayer formation on the outer surface is difficult for such isotherms. Therefore, the common B.E.T. method was not used for determination of the specific surface area of the solid. The specific surface area was determined using non-local density functional theory (NLDFT) method with the model for nitrogen adsorption at 77 K on silica (assuming cylindrical pore) implemented in Quantachrome NovaWin software package.

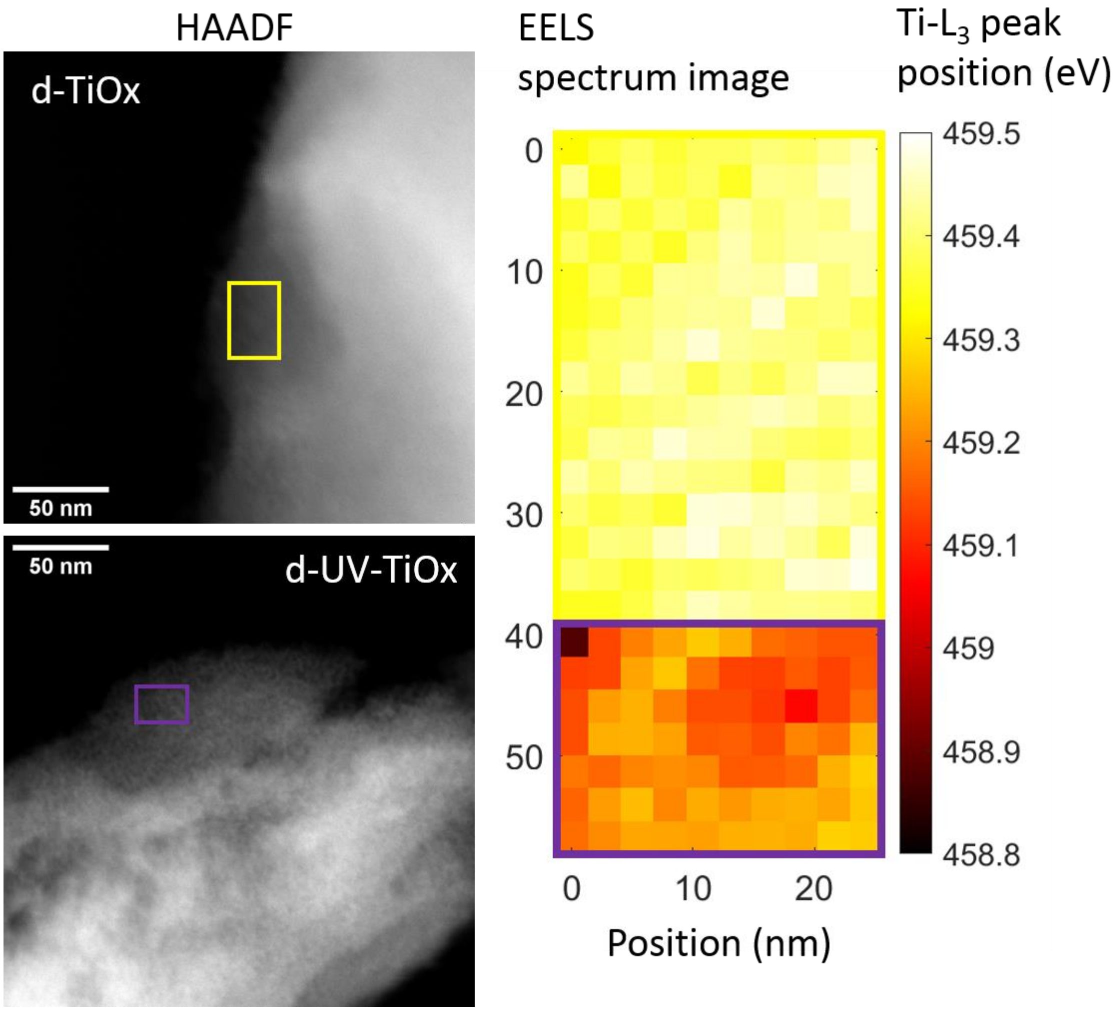

Figure S10 STEM images and EELS spectrum images acquired at d-UV-Ti_xO_y and d-Ti_xO_y in the areas marked with yellow and purple boxes. The pixel size was 2.7 nm to prevent beam damage. Left shows the colour code for the Ti-L_3 position. The spatial variation within each sample is smaller than between them. The spectra given in Figure 6b are averaged over the area shown in yellow and purple.

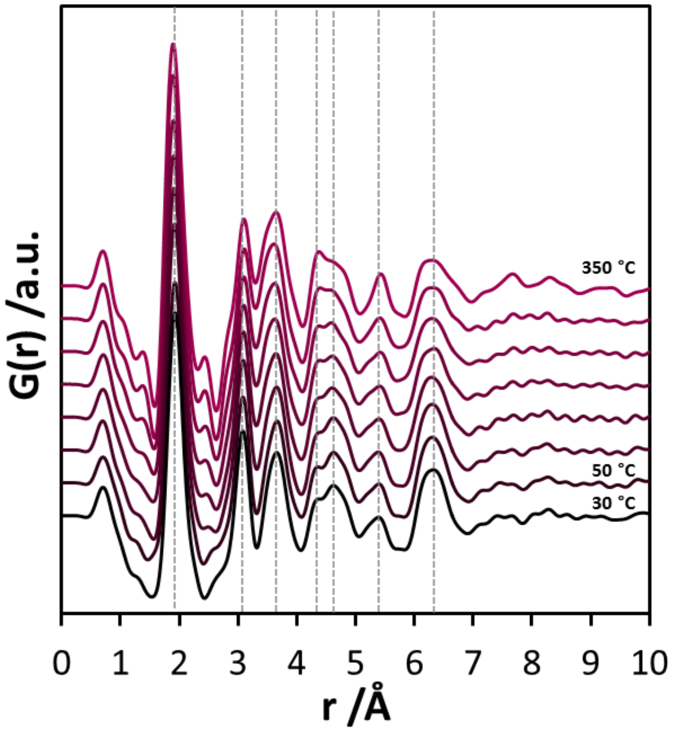

Figure S11 Stack plot of experimental PDFs obtained from *in situ* total scattering data collected from d-Ti_xO_y in the temperature range between 30 - 300 °C.

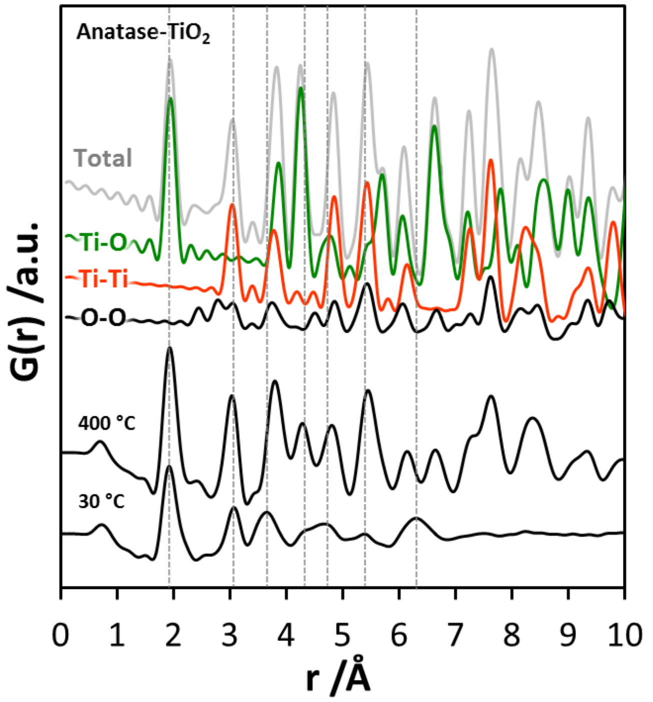

Figure S12 Partial PDFs of the atom pairs calculated from anatase-TiO_2 structure[12] compared to the PDFs collected from d-Ti_xO_y sample at 30 °C and 400 °C.

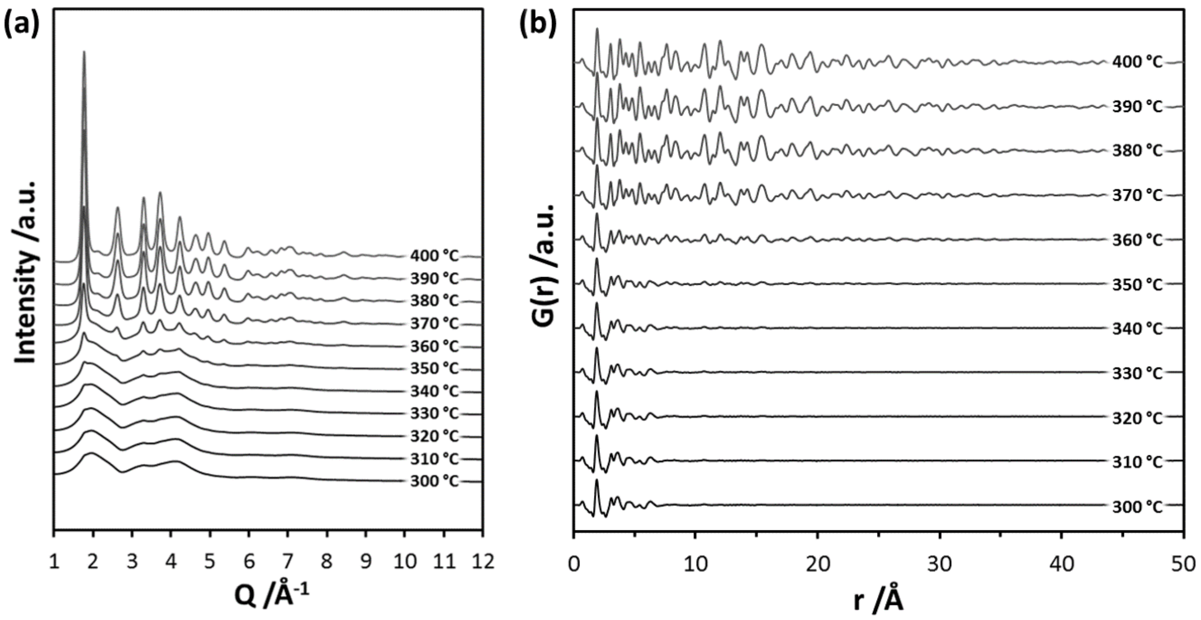

Figure S13 (a) Scattering data and (b) respective long range PDFs obtained from *in situ* temperature-dependent experiments.

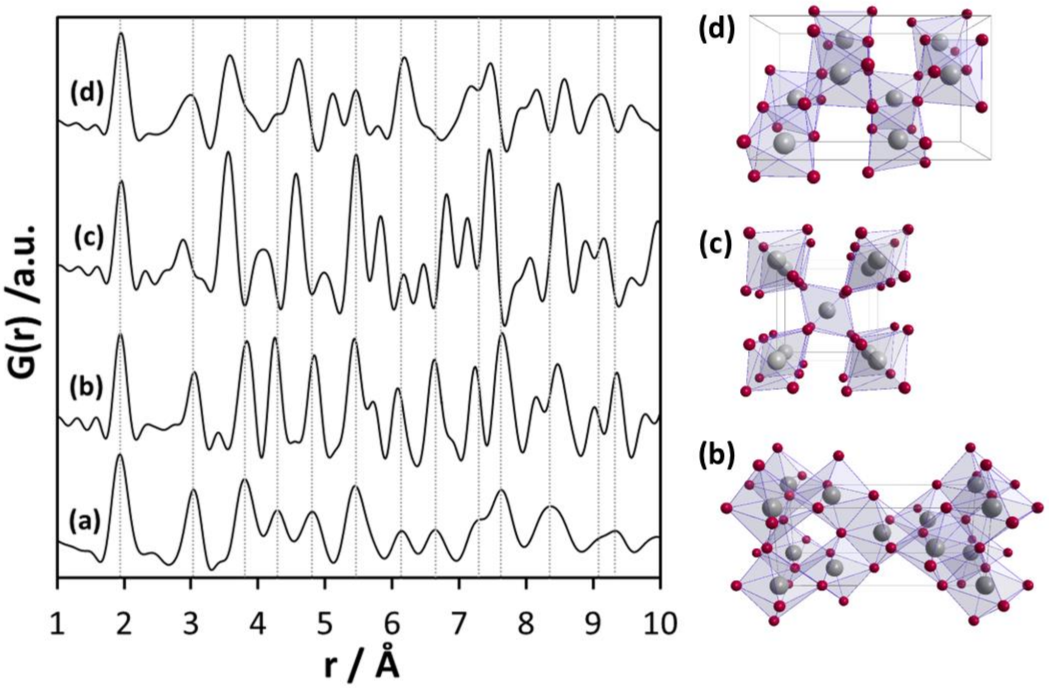

Figure S14 (a) Experimental PDF of d-Ti_xO_y obtained at 400 °C compared to PDFs simulated using (b) anatase,[12] (c) rutile[13] and (d) brookite.[14] PDFs are normalised according to the first Ti-O peak intensity. The corresponding structure motives are shown on the right side.

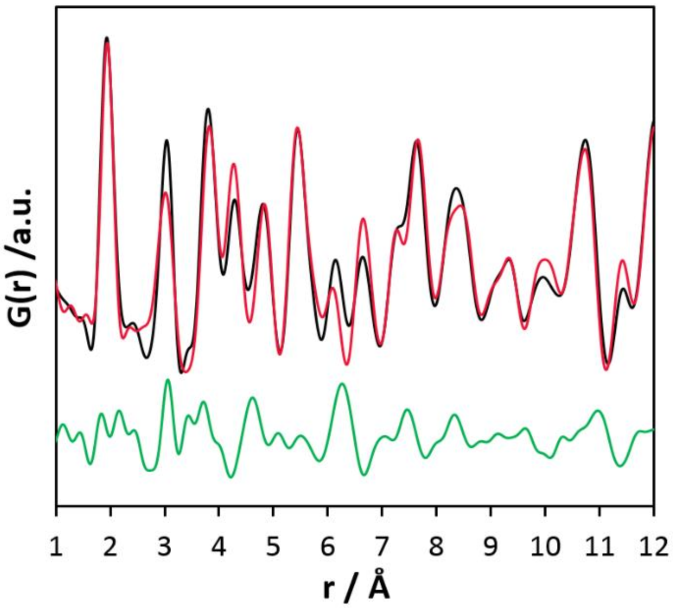

Figure S15 Refinement of the PDF obtained from d-Ti_xO_y at 400 °C using anatase structure.[12] The calculated PDF is plotted in red colour on top of the experimental PDF given in black. Note that the difference (calculated PDF intensity subtracted from that of the experimental PDF) curve is given in green colour with an offset below.

Table S1 Refined parameters for the PDF obtained from d-Ti_xO_y at 400 °C using anatase-TiO_2 structure (q_{max}= 22 $Å^{-1}$, q_{damp}= 0.0375 $Å^{-1}$, q_{broad}= 4.067*10^{-6} $Å^{-1}$, sratio= 1.0, Rw= 0.277266)

Parameter		Initial value	Refined value
**Scale factor**		0.400000	0.313966 (0.024)
**Particle diameter /Å**		50.0000	38.9607
**Quadratic corr. factor**		1.00000	1.18737
**Linear corr. factor**		1.00000	0.554167
**Low r sigma ratio**		1.00000	0.87798 (0.051)
**Lattice parameters /Å**			
	**a**	3.78420	3.80281
	**b**	3.78420	3.80281
	**c**	9.51460	9.46832
**ADPs**			
	**Ti**	0.0050000	0.0110564
	**O**	0.0050000	0.0136242

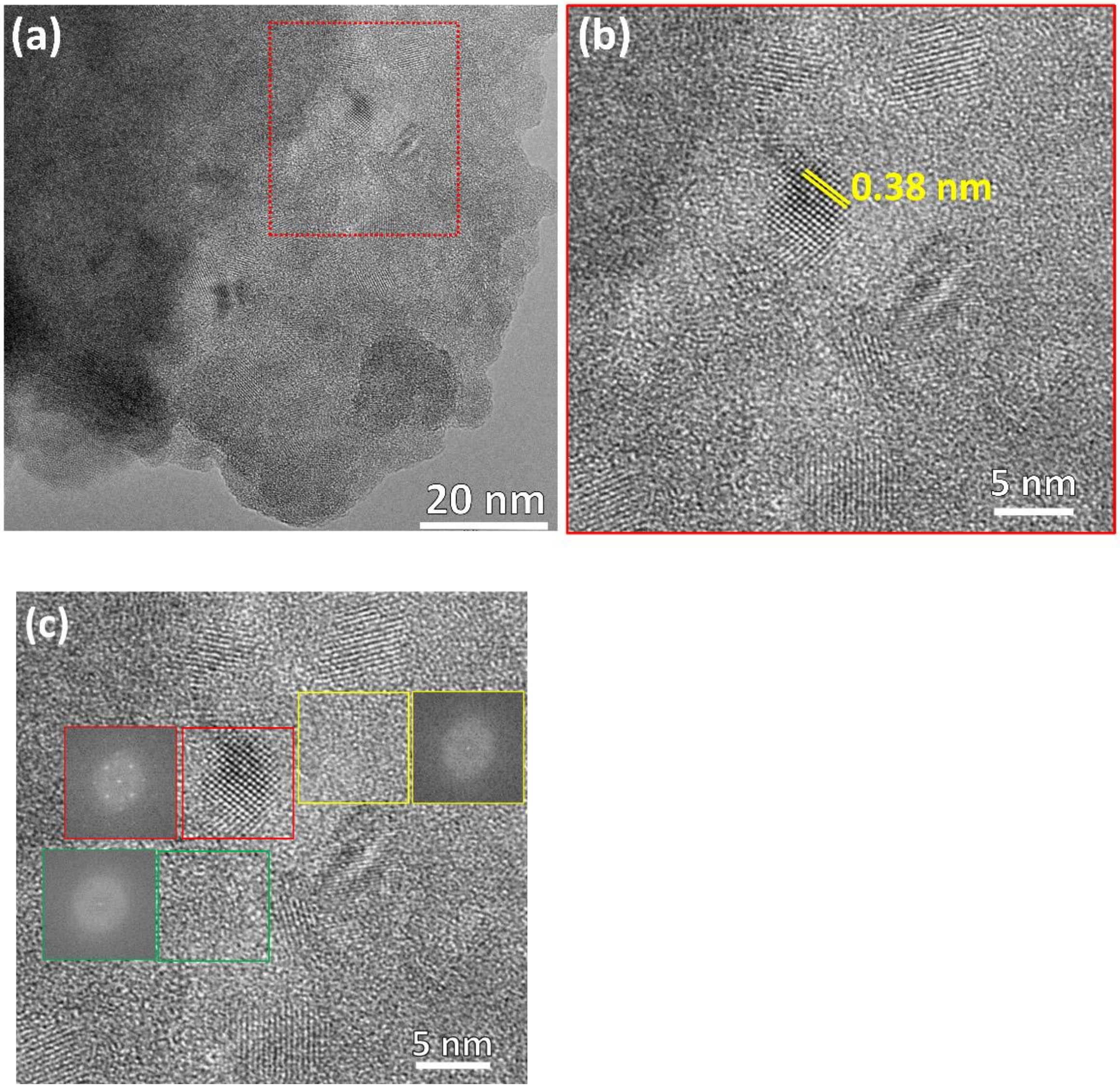

Figure S16 High-resolution transmission electron micrographs obtained after *in situ* temperature dependent total scattering experiments at different magnifications (a,b) and additional electron diffraction patterns (c). Sample was cooled from 400 °C to room temperature.

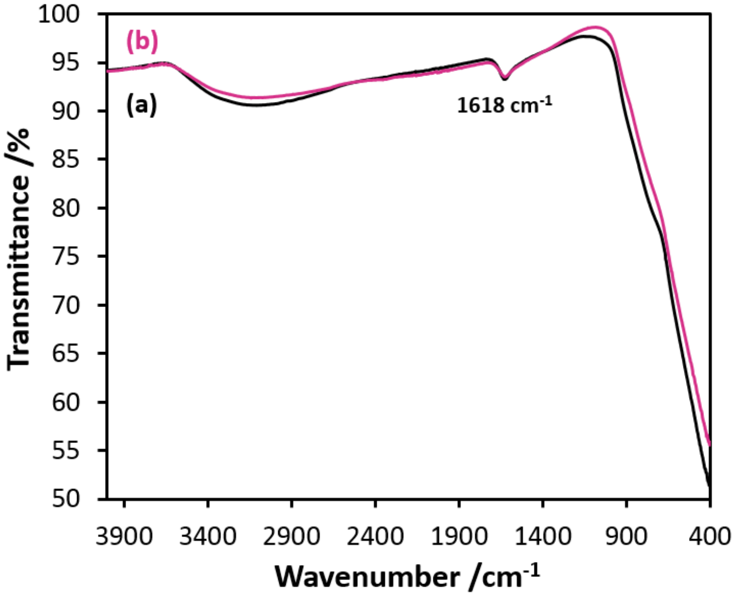

Figure S17 FTIR spectra obtained in ATR mode at room temperature from (a) d-Ti_xO_y and (b) d-Ti_xO_y after heating to 400 °C.

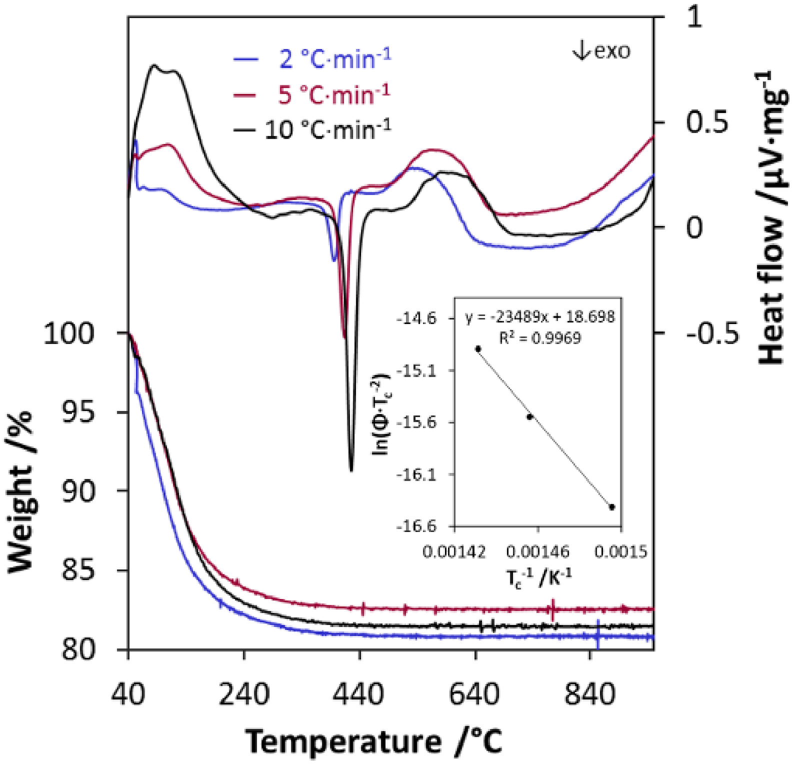

Figure S18 DSC and TG data collected under air flow at heating rates of 2, 5 and 10 °C·min^{-1} (represented by blue, red and black curves respectively) for d-Ti_xO_y. The temperature values corresponding to crystallisation peaks are used to calculate the activation energy for the crystallisation process.[15] Respective Kissinger plot is given as an inset.

References

1. D. Zywitzki, H. Jing, H. Tüysüz, C.K. Chan, *J. Mater. Chem. A* **2017**, *5*, 10957-10967.

2. J. Filik, A.W. Ashton, P.C.Y. Chang, P.A. Chater, S.J. Day, M. Drakopoulos, M.W. Gerring, M.L. Hart, O.V. Magdysyuk, S. Michalik, A. Smith, C.C. Tang, N.J. Terrill, M.T. Wharmby, H. Wilhelm, *J. Appl. Cryst.* **2017**, *50*, 959-966.

3. P. Juhás, T. Davis, C.L. Farrow, S.J.L. Billinge, *J. Appl. Cryst.* **2013**, *46*, 560-566.

4. X. Yang, P. Juhas, C.L. Farrow, S.J.L. Billinge, *ArXiv* **2014**, *1402.3163*.

5. P. Juhás, C.L. Farrow, X. Yang, K.R. Knox, S.J.L. Billinge, *Acta Crystallogr.* **2015**, *A71*, 562-568.

6. L. Farrow, P. Juhás, J. W. Liu, D. Bryndin, E.S. Božin, J. Bloch, T. Proffen, S.J.L. Billinge, *J. Phys.: Condens. Matter* **2007**, *19*, 1-7.

7. S. Zhang, C. Scheu, *Microscopy* **2018**, *67*, i133-i141.

8. M.D. Hanwell, D.E. Curtis, D.C. Lonie, T. Vandermeersch, E. Zurek, G.R. Hutchison, *J. Cheminformatics* **2012**, *4*, 1-17.

9. K. Brandenburg, H. Putz, DIAMOND (Version 3.2k), Crystal Impact GbR, Bonn, Germany, **2005**.

10. D.A. Wright, D.A. Williams, *Acta Cryst.* **1968**, *B24*, 1107-1114.

11. H. Zhang, B. Chen and J.F. Banfield, *Physical Review B* **2008**, *78*, 214106.

12. M. Horn, C. Schwebdtfeger and E. Meagher, *Z. Kristallogr. - Cryst. Mater.* **1972**, *136*, 273-281.

13. E.P. Meagher and G.A. Lager, *Can. Min.* **1979**, *17*, 77-85.

14. W.H. Baur and A.A. Khan, *Acta Cryst.* **1971**, *B27*, 2133-2139.

15. H.E. Kissinger, *J. Res. Nat. Bur. Stand.* **1956**, *57*, 217-221.
